# Solid Lipid Nanoparticles and Nanostructured Lipid Carriers for Anticancer Phytochemical Delivery: Advances, Challenges, and Future Prospects

**DOI:** 10.3390/pharmaceutics17081079

**Published:** 2025-08-21

**Authors:** Shery Jacob, Rekha Rao, Bapi Gorain, Sai H. S. Boddu, Anroop B. Nair

**Affiliations:** 1 Department of Pharmaceutical Sciences, College of Pharmacy, Gulf Medical University, Ajman P.O. Box 4184, United Arab Emirates; 2 Department of Pharmaceutical Sciences, Guru Jambheshwar University of Science and Technology, Hisar 125001, India; rekhaline@gjust.org; 3 Department of Pharmaceutical Sciences and Technology, Birla Institute of Technology, Mesra, Ranchi 835215, India; bapi.gn@gmail.com; 4 Department of Pharmaceutical Sciences, College of Pharmacy and Health Sciences, Ajman University, Ajman P.O. Box 346, United Arab Emirates; s.boddu@ajman.ac.ae; 5 Center of Medical and Bio-Allied Health Sciences Research, Ajman University, Ajman P.O. Box 346, United Arab Emirates; 6 Department of Pharmaceutical Sciences, College of Clinical Pharmacy, King Faisal University, Al-Ahsa 31982, Saudi Arabia; anair@kfu.edu.sa

**Keywords:** anticancer phytochemicals, classification, solid lipid nanoparticles, nanostructured lipid carriers, preparation, in vitro characterization methods, patents

## Abstract

Phytochemicals exhibit a broad spectrum of pharmacological activities, including significant anticancer potential. However, their clinical translation is often hampered by poor aqueous solubility, low bioavailability, and chemical instability. Lipid-based nanocarriers, especially solid lipid nanoparticles (SLNs) and nanostructured lipid carriers (NLCs), have proven to be effective strategies for addressing these challenges. These nanocarriers improve the solubility, stability, and bioavailability of phytochemical-based anticancer agents, while enabling controlled and tumor-specific drug release. Encapsulation of anticancer phytochemicals such as curcumin, quercetin, resveratrol, silymarin, and naringenin in SLNs and NLCs has demonstrated improved therapeutic efficacy, cellular uptake, and reduced systemic toxicity. Co-delivery strategies, combining multiple phytochemicals or phytochemical–synthetic drug pairs, further contribute to synergistic anticancer effects, dose reduction, and minimized side effects, particularly important in complex cancers such as glioblastoma, breast, and colon cancers. This review presents a comparative overview of SLNs and NLCs in terms of formulation methods, in vitro characterization, and classification of key phytochemicals based on chemical structure and botanical sources. The roles of these lipidic carriers in enhancing anticancer activity, challenges in formulation, and recent patent filings are discussed to highlight ongoing innovations. Additionally, hybrid lipid–polymer nanoparticles are introduced as next-generation carriers combining the benefits of both systems. Future research should aim to develop scalable, biomimetic, and stimuli-responsive nanostructures through advanced surface engineering. Collaborative interdisciplinary efforts and regulatory harmonization are essential to translate these lipid-based carriers into clinically viable platforms for anticancer phytochemical delivery.

## 1. Introduction

Plant-derived natural products with diverse pharmacological activities offer numerous health benefits, including disease prevention, support for metabolic and immune health, and promotion of gut health. They are safer, better tolerated, and less likely to cause resistance compared to synthetic drugs. Phytochemicals, derived from plants, play a crucial role in plant survival, such as providing protection against environmental stressors, including pollution, UV radiation, microbial threats, pathogens, and predators, while also regulating growth and reproduction [1]. Their diverse biological activities make them valuable not only to plants but also to human health, where they are widely studied for their medicinal and therapeutic benefits. These include antioxidants and anti-inflammatory agents such as polyphenols, flavonoids, carotenoids, allyl sulfides, curcuminoids, and tannins [2]. Hormone-like phytochemicals include isoflavones from soy (*Glycine max*), lignans from flaxseeds (*Linum usitatissimum*), and coumestans from *Cullen corylifolium* [3]. Several phytochemicals function as enzyme regulators, including protease inhibitors, indole compounds, glucosinolates, and terpenoids, all of which play critical roles in metabolic modulation [4]. Others serve as anti-infective agents, such as proanthocyanidins, alkaloids, lectins, and quinones, contributing to host defense against pathogens [5]. In addition, many exhibit organ-specific therapeutic effects, for instance, neuroprotective agents [6] such as ginsenosides, bacosides, huperzine A, alantamine, and anthocyanins; cardioprotective compounds [7] like quercetin, catechins, and rutin; and immune-modulating agents [8], including beta-glucans, echinacosides, withanolides, and polysaccharides [9] from medicinal mushrooms.

## 2. Phytochemicals with Antitumor Properties

Despite advances in modern medicine, cancer remains a leading cause of death globally and continues to impose a substantial burden on healthcare systems and societies. According to recent data, the global cancer burden reached 19.3 million new cases and nearly 10 million deaths in 2020 [10]. Phytochemicals serve as effective complementary agents in cancer therapy by enhancing the efficacy of conventional chemotherapy, reducing toxicity, and acting through multiple pathways [11]. When combined with conventional chemotherapeutic agents, they promote cancer cell death, inhibit metastasis, and allow for lower chemotherapy doses. Additionally, their antioxidant properties help protect normal tissues from treatment-induced oxidative damage, making them promising candidates in integrative cancer care [12].

The molecular mechanisms driving cancer initiation and progression are diverse and often complex, varying substantially across cancer types. Despite advancements in genomics and epigenetics, many pathways remain incompletely understood. Nevertheless, alterations in tumor suppressor genes, oncogenes, and epigenetic regulators have been widely implicated in carcinogenesis [13]. Conventional pharmacological treatments, including chemotherapy, often fail to achieve long-term remission due to their non-specific toxicity, development of drug resistance, and adverse effects on healthy cells, which collectively limit treatment efficacy and patient compliance [14]. These limitations have spurred growing interest in naturally derived compounds as alternative or adjunct therapeutic agents, given their potential to target multiple signaling pathways with reduced systemic toxicity and improved safety profiles.

Phytochemicals such as resveratrol, curcumin, berberine, vincristine, vinblastine, paclitaxel, epigallocatechin-3-gallate, genistein, camptothecin, quercetin, thymoquinone, betalains, ursolic acid, sulphoraphane, plumbagin, lycopene, and β-Lapachone have gained significant attention in cancer therapy due to their ability to induce apoptosis, inhibit cell growth, suppress angiogenesis, and modulate critical cancer-related signaling pathways, making them promising adjuncts or alternatives to conventional chemotherapeutics.

### 2.1. Reseveratrol

Resveratrol promotes cancer cell death through both p53-mediated apoptosis (via upregulation of Bax, NOXA, and PUMA) and autophagy by activating Sirt1 and AMPK [15]. It suppresses metastasis by inhibiting epithelial–mesenchymal transition through downregulation of TGF-β1/Smads, Wnt/β-catenin, PI3K/Akt/NF-κB, and Gli1 signaling pathways. Additionally, resveratrol impedes angiogenesis by inhibiting VEGF expression through a HIF-1α-dependent mechanism. Resveratrol has been reported to function as an antagonist of estrogen receptor alpha and an agonist of estrogen receptor beta. Notably, its protective effects against prostate cancer have been linked to the upregulation of this estrogen receptor expression [16].

### 2.2. Curcumin

Curcumin modulates multiple cells signaling pathways by targeting secondary messengers like PI3K, Akt, mTOR, STAT3, Wnt/β-catenin, TGF-β, NF-κB, AMPK, and the NLRP3 inflammasome. It also influences oxidative stress by generating reactive oxygen species, increasing intracellular Ca^2+^ levels, reducing mitochondrial membrane potential, and activating caspase-3 [17]. Curcumin promotes apoptosis by upregulating pro-apoptotic Bax, downregulating anti-apoptotic Bcl-2, releasing cytochrome c, and enhancing poly (ADP-ribose) polymerase cleavage [18].

### 2.3. Berberine

Berberine suppresses cancer cell proliferation by modulating the cell cycle, inducing autophagy, and promoting apoptosis [19]. It also inhibits invasion and metastasis by blocking epithelial–mesenchymal transition and downregulating metastasis-associated proteins and signaling pathways. Furthermore, this alkaloid exerts antiproliferative effects through interactions with microRNAs and inhibition of telomerase activity. In various cancer cell types, berberine has been shown to inhibit proliferation by inducing autophagy and to overcome drug resistance by modulating autophagic pathways [20].

### 2.4. Camptothecin

20(S)-Camptothecin acts as a chemotherapeutic agent by reversibly binding to DNA topoisomerase I and its DNA complex, stabilizing the cleavable complex and inducing double-strand DNA breaks during replication and transcription [21]. Recent studies indicate that camptothecin promotes the degradation of the Werner syndrome protein via the ubiquitin-proteasome pathway by altering its cellular localization. The extent of this protein degradation was found to correlate with increased sensitivity of breast cancer cells to this pentacyclic alkaloid, suggesting its potential as a predictive biomarker for this natural compound’s responsiveness [22].

### 2.5. Vincristine, Vinblastine, and Paclitaxel

Plant-derived anticancer agents like vincristine, vinblastine, and paclitaxel (Taxol) exert their effects through diverse mechanisms, including disruption of microtubule dynamics, thereby contributing to the suppression of tumor growth and vascularization. They disrupt signaling pathways that regulate epithelial–mesenchymal transition, preventing cancer cell migration and invasion, promoting carcinogen elimination, blocking mitosis, reducing inflammation, and triggering apoptosis at different cancer stages [23]. Additionally, they contribute to downregulating cellular energetics, promoting invasion and metastasis, stimulating new blood vessel formation, and supporting unlimited replicative potential.

### 2.6. Epigallocatechin-3-Gallate

Polyphenols such as epigallocatechin-3-gallate have been found to inhibit cancer cell proliferation and regulate key apoptosis-related proteins (e.g., Bid, BAX, and Bcl-2), thereby promoting cancer cell death [24]. These compounds also influence metabolic pathways such as glycolysis, the pentose phosphate pathway, the tricarboxylic acid cycle, and serine metabolism, further restricting tumor progression [25].

### 2.7. Genistein

Terpenoids such as genistein suppress angiogenesis, limiting tumor access to nutrients and oxygen [26]. This isoflavone demonstrates anticancer effects through multiple mechanisms, including inhibition of tyrosine kinases, modulation of Hedgehog-Gli1 signaling, regulation of epigenetic modifications, and interference with cell cycle progression as well as Akt and MEK signaling pathways [27]. This plant flavonoid’s anticancer activity is time and dose-dependent and may vary among women depending on physiological factors like menopausal status. Furthermore, phytochemicals affect inflammation indicators, which include heat, swelling, redness, and pain. Collectively, their anti-inflammatory and anticancer properties highlight their potential as both preventive and therapeutic agents.

### 2.8. Quercetin

Quercetin exerts its anticancer effects by modulating multiple signaling pathways within cancer cells, including p53, NF-κB, MAPK, JAK/STAT, PI3K/Akt, and Wnt/β-catenin [28]. It also influences the expression of oncogenic and tumor-suppressor non-coding RNAs, contributing to its therapeutic potential. Moreover, this plant flavonol modulates various intracellular signaling molecules, including TNF-α, Bax, Bcl-2, caspases, and VEGF.

### 2.9. Thymoquinone

Thymoquinone exhibits a range of anticancer activities, including anti-proliferative, pro-apoptotic, antioxidant, cytotoxic, anti-metastatic, and natural killer cell-mediated effects [29]. These actions are driven by its modulation of key molecular mechanisms and signaling pathways, notably p53, NF-κB, and PI3K/Akt. Emerging experimental evidence and recent advances support the potential of this plant pigment as a promising therapeutic agent for inhibiting tumor initiation, progression, and metastasis across various cancer types. This natural compound induces G1 phase cell cycle arrest in human breast, colon, and osteosarcoma cancer cells by inhibiting the activation of cyclin D and cyclin E, while upregulating the cyclin-dependent kinase inhibitors p21 and p27 [30].

### 2.10. Betalains

Betalains, a class of natural compounds (e.g., betanin and betaxanthins), exhibit anti-proliferative and pro-apoptotic activities by triggering caspase activation, altering mitochondrial membrane potential, and modulating key regulatory proteins such as BAX and Bcl-2 [30]. They also contribute to cell cycle arrest and inhibit critical signaling pathways, including PI3K/Akt/mTOR, NF-κB, and p53, with additional potential to suppress tumor angiogenesis.

### 2.11. Ursolic Acid

Ursolic acid is a pentacyclic triterpenoid that exhibits pro-apoptotic, anti-proliferative, anti-metastatic, and anti-angiogenic effects by modulating multiple oncogenic signaling pathways, including p53, Wnt/β-catenin, Ras, NF-κB, TRAIL, and STAT3, highlighting its potential as a multi-targeted therapeutic agent in cancer treatment [31,32]. Additionally, its antioxidant and anti-inflammatory properties indirectly enhance its antitumor potential by reducing oxidative stress and inflammation, which are key contributors to tumorigenesis.

### 2.12. Sulforaphane

Sulforaphane, a natural isothiocyanate derived from glucoraphanin found in cruciferous vegetables like broccoli, exhibits significant anticancer properties. It exerts its effects through multiple mechanisms, including activation of the Nrf2 pathway, which induces phase II detoxifying and antioxidant enzymes, and inhibition of histone deacetylases, non-coding RNAs, and DNA methyltransferases, leading to favorable epigenetic changes [33]. Sulforaphane also promotes apoptosis, induces cell cycle arrest at the G2/M phase, and inhibits inflammation, angiogenesis, and metastasis by targeting key molecules such as NF-κB, COX-2, VEGF, and mitochondrial membrane potential [34].

### 2.13. Plumbagin

Plumbagin induces cell cycle arrest (G2/M phase) and triggers mitochondrial-mediated apoptosis by elevating reactive oxygen species levels and disrupting mitochondrial membrane potential [34]. Additionally, plumbagin suppresses cancer cell proliferation, invasion, and metastasis through modulation of signaling pathways like NF-κB, STAT3, PI3K/Akt/mTOR, and interactions with ATM-p53 DNA-damage response pathways [35].

### 2.14. Lycopene

Literature reviews highlight lycopene’s potent singlet oxygen-quenching antioxidant properties, induction of phase II detoxifying enzymes, promotion of apoptosis, inhibition of cell proliferation and cancer cell cycle progression, as well as modulation of cellular communication and signaling pathways [36]. In addition, lycopene’s ability to inhibit the PI3K/Akt pathway and activate apoptosis in lung cancer models has been reported [37].

### 2.15. β-Lapachone

β-Lapachone demonstrates potent anticancer effects, particularly in tumors overexpressing NQO1. Upon bioactivation by NQO1, β-Lapachone undergoes futile redox cycling, leading to bursts of reactive oxygen species and DNA damage, resulting in selective cancer cell death [38]. Preclinical research also demonstrates its potential to inhibit the PI3K/Akt/mTOR pathway, suppress angiogenesis, and reduce metastasis in cervical cancer models [39].

A comprehensive overview of the major classes of anticancer phytochemicals highlighting representative compounds, their key botanical sources, physicochemical properties, and major formulation challenges related to pharmaceutical or therapeutic applications is summarized in Table 1.

## 3. Oral Bioavailability and Stability Issues

Oral drug delivery is the most preferred and widely accepted route due to its simplicity, noninvasive nature, suitability for long-term and repeated administration, ease of scaling up, and excellent patient compliance [40]. Oral administration of phytochemicals, known for their diverse pharmacological activities, demonstrates significant pharmacokinetic and biopharmaceutical challenges that hinder their clinical application. These compounds are vulnerable to enzymatic degradation in the gastrointestinal tract, and their polar nature and molecular size limit their ability to permeate the blood–brain barrier, endothelial layers, and mucosal tissues [41,42]. Additionally, poor water solubility, rapid metabolism, low systemic bioavailability, chemical instability, and short biological half-life further impede their clinical utility [43]. For instance, berberine shows promising anticancer activity; however, its clinical effectiveness is hindered by poor water solubility, rapid metabolism, and low intestinal absorption [19]. Consequently, designing nanoformulations that enhance their gastrointestinal uptake holds significant potential to improve their therapeutic impact against cancer [44].

Quercetin possesses strong therapeutic potential but faces poor bioavailability due to its low solubility, limited permeability, and extensive first-pass metabolism. It is also chemically unstable, being susceptible to oxidation, hydrolysis, and photodegradation under physiological and environmental conditions. Recently, beta-cyclodextrin-capped self-assembled zein nanoparticles have been reported as a stable delivery system for this natural compound [45].

Despite their therapeutic potential, many phytochemicals are often limited by their low aqueous solubility and reduced bioavailability, which restricts their clinical application. As a result, higher doses are often required to achieve therapeutic effects, raising concerns about cost and increasing the risk of toxicity to healthy tissues and peripheral organs [46]. Inter-individual differences in pharmacokinetics and significant first-pass metabolism also led to variable therapeutic outcomes, as observed with resveratrol, extensively metabolized in the intestine and liver, resulting in low and inconsistent systemic bioavailability among individuals [47,48].

Oral phytochemical delivery is further challenged by gastrointestinal tract viscosity, dietary factors, and enzymatic metabolism, which can alter the bioactivity of phytochemicals [49,50,51]. Phytochemical stability in solid and aqueous formulations is governed by various external, and physicochemical factors [52]. In the solid state, moisture can promote hydrolysis, especially in compounds containing ester or amide bonds, whereas elevated thermal conditions accelerate degradation by increasing molecular collision frequency. Curcumin, which contains β-diketone and enol groups, exhibits poor stability in the presence of moisture and elevated temperatures due to its susceptibility to hydrolysis and oxidation [53].

Certain flavonoids and phenolic compounds [54] show thermal sensitivity, remaining stable at low temperatures ~4 °C) but degrading rapidly at higher temperatures (40 °C) [55,56]. Carotenoids are vulnerable to structural changes and oxidative damage during thermal treatment due to their conjugated double bonds [57], while UV light and oxygen further promote oxidative degradation [58,59]. For instance, astaxanthin, a keto-carotenoid with notable antitumor properties, undergoes significant thermal degradation, losing over 10% of its all-trans form at just 70 °C within 1 h and up to ~30% after 16 h, highlighting the challenge of preserving its bioactivity during processing [60].

The surface pH changes or different polymorphic forms can impact stability [61]. Curcumin’s stability can decrease sharply when exposed to acidic or alkaline environments due to ionization and degradation [26]. Additionally, curcumin’s poorly soluble crystalline polymorphs are more prone to degradation than its amorphous counterparts.

Smaller particle sizes and incompatible excipients increase susceptibility to degradation, as do residual solvents and trace metals [56,62]. In aqueous solutions, stability is primarily influenced by pH, temperature, light, and oxygen. Phenolic compounds degrade more at neutral pH, while flavan-3-ols are more stable in acidic environments [57,63,64]. Thymoquinone, although highly soluble in water, is unstable under light and pH fluctuations, making pure aqueous systems unsuitable for its delivery [65]. Stability is further impacted by factors such as solvent composition, ionic strength, and the presence of impurities or trace metal ions [66]. At high concentrations, phytochemicals may also aggregate or precipitate, compromising their effectiveness [62]. These limitations highlight the pressing need for advanced delivery systems to improve the bioavailability, safety, and site-specific efficacy of phytochemicals.

## 4. Nanoparticle-Based Approaches for Phytochemical Delivery

Oral delivery of phytochemicals is limited by physicochemical constraints such as poor water solubility and instability, and biopharmaceutical barriers including rapid metabolism and low membrane permeability. Various advanced delivery approaches, such as nanoparticles [67], vesicular nanocarriers [68], and self-nanoemulsifying systems [69], have been explored to address the bioavailability and stability challenges of phytochemicals.

Nanosystems serve as highly efficient delivery platforms due to their submicron dimensions and unique physicochemical properties, which enhance solubility and stability, and facilitate passive or active targeting of active compounds to specific tissues and cells [70]. These capabilities have been demonstrated across diverse therapeutic areas, including oncology, infectious diseases, neurology, and inflammation, and are increasingly being explored in clinical settings [71]. These nanocarriers can exist in various forms, such as particulate, soluble, or conjugated with targeting ligands, enabling them to embed hydrophilic, hydrophobic, or amphiphilic drugs while protecting them from enzymatic degradation or early clearance by the reticuloendothelial system. Nanoparticles are fabricated using a wide array of natural, semi-synthetic, or synthetic polymers and lipids, through diverse manufacturing techniques [42]. These systems are commonly formulated as aqueous dispersions or alternatively incorporated into semisolid gels, transdermal films, or ocular inserts to enhance residence time and improve patient compliance.

A diverse range of nanocarrier systems has been engineered to boost the therapeutic efficacy of bioactive compounds especially phytochemicals by enhancing their solubility, stability, and bioavailability. Each nanocarrier type offers distinct physicochemical properties, formulation flexibility, and biological performance. A comparative overview of these systems based on key attributes composition, encapsulation efficiency, stability, biocompatibility, targeting ability, scale-up potential, and applications, is essential to rationalize their selection for specific therapeutic purposes (Table 2).

For instance, nanoemulsions, while useful for improving solubility, suffer from thermodynamic instability, susceptibility to Ostwald ripening, and limited control over drug release profiles [72]. Liposomes and niosomes, characterized by bilayer structures, provide excellent versatility in encapsulating both hydrophilic and lipophilic drugs. Liposomes are composed of natural or synthetic phospholipids and are widely explored for targeted drug delivery, while niosomes utilize non-ionic surfactants, offering better stability and cost-effectiveness Effect [73,74]. Liposomes, although highly biocompatible, are prone to rapid clearance by the reticuloendothelial system, have limited loading capacity for hydrophobic phytochemicals, and require complex manufacturing processes [75,76]. Niosomes, despite their stability and cost-effectiveness, may suffer from limited drug loading capacity and potential vesicle aggregation over time, affecting long-term stability and scalability [77]. Cubosomes and transferosomes exhibit unique advantages in terms of controlled and enhanced transdermal delivery, owing to their deformable structures and internal architecture [78]. Cubosomes may encounter issues such as drug leakage during in vivo transit and potential cytotoxicity related to commonly used surfactants like Pluronic F127, which may limit their clinical translation. Ivosomes, which combine phospholipids and ionic liquids to enhance drug solubility and permeability, are limited by potential cytotoxicity stemming from ionic liquid components and formulation instability, posing challenges for clinical translation [79]. Despite their advantageous deformability, transferosomes are chemically unstable, prone to oxidative degradation, and susceptible to aggregation or drug leakage during storage, compounded by complex and costly manufacturing processes [80]. In contrast, ethosomes and transethosomes incorporate high ethanol concentrations, enhancing skin permeation and fluidity of the vesicle membrane. Ethosomes and transethosomes, though effective in enhancing skin permeation, face limitations such as physical instability, ethanol-induced vesicle disruption, burst drug release, and scale-up challenges, which can hinder their clinical translation [81,82]. Polymeric nanoparticles, particularly those made from PLGA, PCL, or chitosan, allow for tunable drug release and strong structural stability, though challenges remain in achieving high drug loading, burst release, costly and scalable production [83]. Dendrimers, with their well-defined architecture and multivalency, enable precise drug conjugation, high drug loading, and targeted delivery. However, despite these advantages, they are limited by cytotoxicity at higher generations, complex synthesis, and high production costs, which can hinder their large-scale pharmaceutical application [84].

Inorganic nanoparticles, including gold, iron oxide, and mesoporous silica nanoparticles, offer unique imaging and therapeutic functionalities (theranostics) but often face challenges in biodegradability and long-term safety [85,86]. Metallic nanoparticles, though valuable in theranostics, carry the risk of oxidative stress, DNA damage, and organ toxicity, making them less favorable for long-term or phytochemical-based therapies [87]. Polymeric nanoparticles, though offering controlled release, may exhibit reduced biocompatibility, higher production costs, and potential cytotoxicity depending on the polymer used [88]. Dendrimers offer high drug loading and functionalization potential but are associated with significant cytotoxicity and high cost of synthesis [89]. Carbon nanotubes demonstrate promising drug loading and targeting capabilities, yet concerns about long-term toxicity, poor biodegradability, and immunogenicity limit their clinical application [90]. Polymeric micelles self-assembled from amphiphilic block copolymers are excellent carriers for poorly soluble anticancer phytochemicals, offering improved circulation, passive targeting via the EPR effect, and the potential for stimulus-responsive drug release in tumor microenvironments [91]. Polymeric micelles can potentially enhance the solubility of hydrophobic phytochemicals but often show poor stability in vivo due to dilution below their critical micelle concentration, leading to premature drug release [92]. However, they face notable limitations: they can dissociate upon dilution in blood, leading to premature drug release, exhibit relatively low physical stability, often have modest encapsulation efficiency, and typically provide limited control over drug release kinetics.

In cancer therapy, programmable lipid nanoparticles can deliver chemotherapeutics or genetic payloads in a controlled manner, bypassing physiological barriers like the blood–brain barrier. For instance, a dual peptide-functionalized nanocarrier was developed to deliver IRAK4 inhibitors across the blood–brain barrier and target microglia in the hypothalamus, and was tested in mouse models of acute and chronic neuroinflammation [93]. Nanotechnology significantly advances phytochemical delivery by improving their stability, solubility, and targeting, while also enabling integrated diagnostic and therapeutic applications, marking a major shift in clinical phytomedicine. Among these nanocarrier systems, surface modification with ligands such as folic acid, peptides, or antibodies greatly enhances their targeting efficiency. In terms of scalability, lipid- and polymer-based systems offer superior translational potential, whereas complex hybrid or inorganic formulations often face greater challenges in large-scale production.

SLNs and NLCs are ideal for delivering anticancer phytochemicals due to their superior physical stability, controlled release, biocompatibility, and high drug-loading capacity. They outperform nanoemulsions, vesicular carriers, inorganic nanoparticles, and polymeric micelles in protecting labile compounds and minimizing toxicity.

## 5. Functionalization of Nanoparticles

The physicochemical characteristics of the nanocarrier system such as particle size, surface potential, hydrophilicity, and surface functionalization along with the selection of encapsulated phytochemical and matrix materials, play a crucial role in determining the pharmacokinetic profile, permeability across membrane barriers, release kinetics, mucoadhesion properties, and target-specific efficiency [94]. Stimuli-responsive nanocarriers, tailored to internal (pH, redox, enzymatic) or external (temperature, magnetic field, ultrasound) triggers, enhance targeted drug delivery by improving site-specific retention, cellular uptake, and overall bioavailability [95]. For example, magnetic iron-oxide nanoparticles have been used to deliver quercetin to tumor sites under external magnetic guidance, improving accumulation and reducing off-target toxicity [96].

Gold-based systems, such as curcumin-coated plasmonic nanogels, have enabled combined delivery and near-infrared photothermal therapy, enhancing anticancer efficacy [97]. Moreover, smart stimuli-responsive nanocarriers designed to respond to tumor-specific triggers (e.g., pH, enzymes, temperature) have successfully delivered phytochemicals with controlled release profiles and improved tumor targeting [98].

Ligands used for functionalization include antibodies, aptamers, peptides, and small molecules [99]. Triptolide, a potent anticancer diterpenoid extracted from *Tripterygium wilfordii* has been successfully conjugated to a nucleic acid aptamer (AS1411) for targeted delivery in triple-negative breast cancer models [100]. The AS1411-conjugated triptolide complex leverages the aptamer’s high specificity to nucleolin, which is overexpressed on cancer cell surfaces. This functionalization enhances targeted uptake, yields controlled release in response to the acidic tumor microenvironment, and achieves impressive tumor growth suppression with minimal systemic toxicity.

Active targeting offers a promising approach to overcome multidrug resistance as resistance-related proteins like P-glycoprotein are unable to expel drugs delivered through nanoparticle-mediated endocytosis [70]. Taccalonolides are anticancer phytochemicals that stabilize microtubules and retain efficacy against P-glycoprotein–mediated multidrug-resistant cancer cells, as they evade recognition and expulsion by the efflux pump, unlike taxane-based drugs [101]. By leveraging nanoparticle-mediated endocytosis to deliver taccalonolides intracellularly, active targeting further ensures these agents bypass P-gp efflux, offering a promising strategy against drug-resistant cancers. Given the high expression of folate receptors in various malignant tumors and their minimal presence in normal tissues, folic acid-functionalized nanoparticles loaded with curcumin are anticipated to selectively target cancer cells [102]. Lactoferrin-conjugated betulinic acid nanoparticles were developed to overcome the poor solubility and limited cellular uptake of betulinic acid for targeted treatment of aggressive triple-negative breast cancer [103]. These nanoparticles effectively inhibited cell proliferation and viability, inducing cell cycle arrest.

It was reported that SLNs, NLCs significantly enhanced drug delivery to tumors via passive and active targeting mechanisms. Additionally, they improved brain permeability, supporting their potential for targeted therapies in central nervous system disorders [104]. Modulating the physicochemical characteristics of nanocarriers such as SLNs, NLCs, nanoemulsions, polymeric nanoparticles, polymeric micelles, liposomes, dendrimers, carbon nanotubes, and metallic nanoparticles can significantly enhance the therapeutic efficacy of anticancer phytochemicals, minimize systemic toxicity, overcome multidrug resistance, and promote targeted accumulation at tumor sites [105,106].

Chitosan is a safe, biodegradable biopolymer with strong mucoadhesive and antitumor properties, supported by its stable, polycationic structure and broad tissue compatibility, making it ideal for different pharmaceutical applications [107]. In a recent study, chitosan-coated SLNs loaded with *Aloe perryi* extract exhibited significant anticancer activity against A549, LoVo, and MCF-7 cell lines, with IC_50_ values of 11.42 ± 1.16, 16.97 ± 1.93, and 8.25 ± 0.44 μg/mL, respectively, suggesting their potential as effective ALP-based anticancer delivery systems [108]. Lipid nanoparticles were functionalized with mucoadhesive polymers, such as chitosan and its derivatives, to facilitate prolonged oral delivery of phyto-bioactives and reduce premature release in the acidic conditions of the stomach [109]. Further modifications, like grafting chitosan with functional groups (e.g., trimethyl chitosan and lipid conjugates such as palmitic acid), improved mucoadhesion, target-specific delivery, and controlled release.

In summary, functionalization of lipid-based nanoparticles such as SLNs and NLCs is practically feasible and scalable due to their composition from physiologically acceptable lipids and surfactants, many of which are already approved by regulatory agencies. These systems allow surface modification with targeting ligands, enabling active targeting of tumor tissues while maintaining biocompatibility and biodegradability. Compared to other nanosystems, lipid nanoparticles offer simpler formulation processes, lower toxicity, and better compatibility with lipophilic anticancer phytochemicals such as curcumin and resveratrol, making them particularly suitable for clinical translation.

## 6. Lipid-Based Nanoparticles

**Figure 1:**
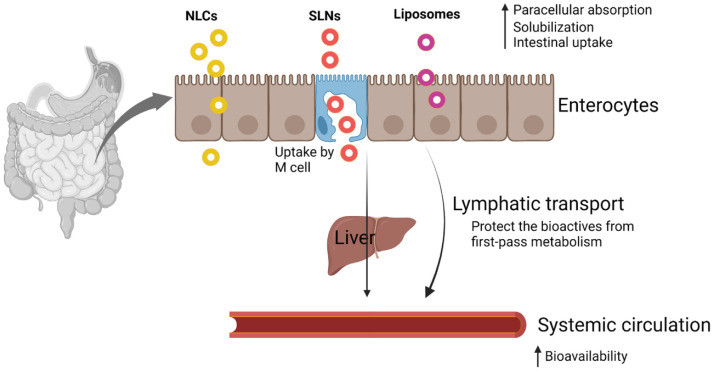
Transport mechanism of lipid-based delivery systems. Reproduced from Ref. [119].

Lipid-based nanoparticles such as SLNs and NLCs are increasingly preferred for the delivery of anticancer phytochemicals due to their excellent biocompatibility, biodegradability, ability to encapsulate poorly water-soluble compounds, and potential for both passive and active targeting [110]. SLNs and NLCs offer significant advantages in the delivery of natural compounds, providing a stable foundation that overcomes many of the limitations associated with other nanocarriers in phytochemical delivery [111]. Typical transport mechanisms of lipid-based nanosystems are presented in Figure 1. SLNs and NLCs enhance the solubility and oral bioavailability of poorly water-soluble compounds by providing a lipid-based matrix that promotes absorption and protects the active ingredients from degradation caused by light, oxygen, moisture, and enzymatic activity [112]. These nanoparticles enable controlled and sustained drug release, enhance cellular uptake due to their nanoscale size, and exhibit biocompatibility, making them appropriate for multiple routes of administration [113,114]. Compared to SLNs, NLCs incorporate both solid and liquid lipids, which increases drug loading capacity and prevents drug expulsion during storage [115]. Additionally, lipid nanoparticles can promote lymphatic uptake, thereby bypass hepatic first-pass metabolism and further enhancing systemic bioavailability. Lipid-based nanomedicines are at the forefront of nanotechnology’s clinical translation, representing the majority of approved nano-delivery systems. They are widely used in cancer therapy, infectious diseases, pain management, and gene delivery [116,117]. Inhalable lipid-based nanoformulations derived from natural products demonstrate significant promise in the treatment of pulmonary diseases due to a range of advantages, including bypassing the hepatic first-pass metabolism, enabling rapid onset of action, and achieving high bioavailability [118]. These systems allow for localized drug delivery, resulting in high pulmonary drug concentrations at lower systemic doses, thereby minimizing systemic side effects and enhancing therapeutic efficacy. Moreover, the lipidic composition can improve drug solubility and stability, facilitate mucosal penetration, and provide sustained drug release.

## 7. Solid Lipid Nanoparticles (SLNs)

**Figure 2:**
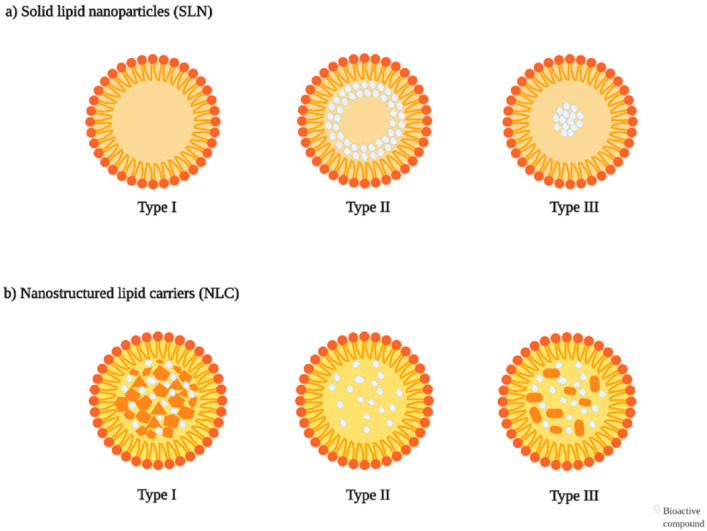
Various types of SLNs and NLCs. Reproduced from Ref. [124].

SLNs, colloidal drug delivery systems composed of nano-sized (10–1000 nm) lipid particles, are conventionally prepared by dispersing a solid lipid matrix in an aqueous phase containing a surfactant as a stabilizing agent [115]. SLNs have received significant attention in drug delivery systems owing to their numerous advantages, including improved physical and chemical stability, high drug loading capacity, customizable drug release profiles, potential for targeted delivery, enhanced bioavailability of poorly water-soluble drugs, excellent biocompatibility, and ease of sterilization [113,120]. SLNs offer enhanced protection for sensitive lipophilic drugs by preventing degradation due to the immobilization of these agents within the solid lipid matrix [121]. Additionally, they demonstrate great potential for administration through various routes such as oral, parenteral, ocular, pulmonary, nasal, rectal, transdermal, and vaginal delivery [122]. Moreover, SLNs can be efficiently produced on a large scale and scaled up using techniques such as high-pressure homogenization, hot-melt extrusion, and ultrasonication, facilitating their potential for commercialization [123]. The three varieties of SLNs are Type I (homogeneous matrix), Type II (drug-enriched shell), and Type III (drug-enriched core) according to their internal structure (Figure 2). The release characteristics and applicability of these categories vary from one another, and NLCs in medication dispersion.

The primary limitations of SLNs include limited drug loading capacity, the risk of drug expulsion during storage, and issues such as particle growth, aggregation, solidification, and polymorphic transitions, all of which are attributed to the crystalline nature of the solid lipid matrix [125]. A significant drawback is the initial burst release, particularly for hydrophilic drugs, as they tend to remain adsorbed on the nanoparticle surface rather than being incorporated into the lipid core. As polar drugs tend to localize mainly within the outer surfactant layer, their loading capacity is inherently limited. To overcome this challenge, lipid–drug conjugates formed by chemically linking drugs to lipoidal molecules like fatty acids or phospholipids have been developed to improve drug incorporation and minimize leakage [126].

In addition, drug release from SLNs is significantly influenced by the distribution of the drug within the lipid matrix, which is governed by the physicochemical properties of the drug, the type of lipid used, and the production conditions. The drug can be located in three primary positions within the SLN structure: (i) homogeneously dispersed within the lipid core, (ii) localized near the surface, or (iii) enriched in the outer shell. Each distribution pattern affects the drug release kinetics differently—surface-localized drugs typically result in burst release, whereas matrix-embedded drugs facilitate sustained release profiles [127,128].

The polymorphic state and crystallinity of the lipid matrix also play a critical role. Lipid matrices with high crystallinity tend to expel the drug during storage (due to lipid rearrangement into more stable polymorphs), whereas less-ordered matrices (e.g., nanostructured lipid carriers) can better retain the drug and control its release [129]. Furthermore, production methods, such as high-pressure homogenization and ultrasonication, affect drug entrapment location due to rapid lipid solidification and surfactant migration, thereby impacting the final release behavior. Understanding these distribution patterns is crucial for tailoring drug release profiles to meet specific therapeutic needs, especially for phytochemicals, where controlled release improves stability, bioavailability, and pharmacokinetic profiles.

The drug release behavior and long-term stability of SLNs are closely influenced by the thermodynamic and crystallographic characteristics of the lipid matrix. Lipids used in SLN formulations undergo polymorphic transitions typically between α (metastable), β′ (intermediate), and β (most stable) forms. These polymorphic forms differ in their packing density and crystal lattice arrangement, which in turn affect drug incorporation capacity and release profile [127,130]. In the α form, the lipid matrix exhibits a loosely packed structure that allows higher drug incorporation but is thermodynamically unstable and prone to transition to the more stable β′ or β forms during storage. This reorganization of the lipid lattice can lead to drug expulsion or redistribution, negatively impacting controlled release and shelf life. The β′ form offers moderate stability and drug retention, whereas the β form, despite its superior thermodynamic stability, is often associated with lower drug-loading efficiency due to its highly ordered and tightly packed structure [131]. To study these transitions and their implications, Differential Scanning Calorimetry (DSC) and X-ray Diffraction (XRD) techniques are widely employed. DSC provides insights into melting behavior, crystallinity index, and enthalpy changes, which reflect the physical state of the lipid and the extent of drug-lipid interaction. XRD complements this by identifying crystalline phases and assessing whether the drug is present in an amorphous or crystalline form within the lipid matrix [132,133]. Such thermal and structural evaluation is crucial for optimizing SLN formulations intended for controlled drug delivery, especially for phytochemicals, which are often sensitive to recrystallization-induced degradation or burst release.

Despite these drawbacks, SLNs play a crucial role in drug delivery by enabling sustained and targeted release, improving permeability across biological barriers, and safeguarding drugs against enzymatic degradation [134]. They are particularly valuable for improving the therapeutic efficacy of poorly soluble drugs and enabling combination therapy for complex diseases. With ongoing research and advancements, SLNs continue to be a promising platform for pharmaceutical applications, including cancer therapy, neurodegenerative disorders, and infectious diseases.

### 7.1. Structural Components of SLNs

The fundamental structural components of SLNs are classified into solid lipids, emulsifiers, and other critical excipients, including co-surfactants, cryoprotectants, charge modifiers, penetration enhancers, targeting ligands, and polymer coatings. The structural integrity of SLNs primarily relies on the selection of solid lipids, which play a crucial role in drug encapsulation, stability, and controlled release. The most commonly used solid lipids include triglycerides (e.g., tristearin, tripalmitin, trilaurin) that offer excellent biocompatibility and sustained drug release properties [135,136]. Partial glycerides such as glyceryl monostearate and glyceryl monooleate are frequently utilized due to their amphiphilic nature, enhancing drug solubility and stability. Fatty acids like stearic acid, palmitic acid, and oleic acid contribute to SLN stability and influence the drug release profile. Fatty acid esters, including glyceryl behenate (Compritol 888 ATO) and glyceryl palmitostearate (Precirol ATO 5), are used for their controlled release capabilities and compatibility with a wide range of drugs. Steroids, such as cholesterol, are incorporated to enhance membrane rigidity and drug entrapment efficiency, particularly for hydrophobic drugs. Additionally, waxes like cetyl palmitate and carnauba wax provide a highly crystalline lipid matrix, ensuring prolonged drug release and better formulation stability.

The choice of solid lipids significantly impacts the physicochemical properties, encapsulation efficiency, and therapeutic performance of SLNs. A major factor influencing drug dispersibility within the lipid matrix of SLNs is the drug’s high lipophilicity, typically characterized by a log P value greater than 2. Ideal phytochemical candidates for lipid formulations are typically neutral or basic, possess polar functional groups, have a low melting point (<150 °C), and exhibit sufficient solubility in both lipids and water [137]. The cytotoxicity of SLNs varied based on the cationic lipid used, with cetyltrimethylammonium bromide being highly toxic at low concentrations (IC_50_ < 10 µg/mL), while dimethyldioctadecylammonium bromide exhibited significantly lower toxicity (IC_50_: 284.06–869.88 µg/mL) after 48 h [138]. SLNs can influence various cell signaling pathways, highlighting the need for thorough biocompatibility and cytotoxicity assessments of empty SLNs [139].

Surfactants form a compact, flexible, and mechanically robust monolayer at the lipid–water interface, preventing particle aggregation and enhancing long-term stability during manufacturing and storage [140]. The choice of surfactants depends on several key factors, including their hydrophilic-lipophilic balance (HLB), biodegradability, cytocompatibility, effect on lipid crystallinity and polymorphism, as well as their influence on particle size [141]. Non-ionic surfactants, such as Tweens, poloxamers, and tyloxapol, are preferred for their biocompatibility and capbility to inhibit nanoparticle aggregation. Anionic surfactants, including sodium lauryl sulfate, sodium cholate, and sodium glycocholate, provide electrostatic repulsion, reducing particle aggregation [142,143,144]. Cationic surfactants like cetyltrimethylammonium bromide or dimethyldioctadecylammonium bromide enhance drug uptake by interacting with negatively charged cell membranes, making them particularly useful for ocular and gene delivery applications [145]. Amphoteric surfactants, such as soybean and egg lecithin, improve biocompatibility and permeability. Additionally, co-surfactants like polyvinyl alcohol, butanol, propylene glycol, and polyethylene glycol (PEG) support formulation stability, regulate particle size and prevent drug leakage [146]. Therefore, the careful selection of surfactants and co-surfactants is essential for optimizing the stability of SLNs, controlling drug release profiles, and enhancing therapeutic efficacy.

Cryoprotectants prevent the physical and chemical degradation of SLNs during freeze-drying and prolonged storage by preventing particle aggregation and maintaining structural integrity [147]. These agents aid in maintaining particle size and structural stability during freezing, drying stress, and subsequent rehydration. Commonly used cryoprotectants include mannitol, trehalose, sucrose, glucose, and sorbitol. Charge modifiers adjust the surface charge of SLNs, influencing stability, cellular uptake, and drug targeting. By preventing aggregation through electrostatic repulsion, they enhance formulation stability and interaction with biological membranes [148]. Cationic charge modifiers like stearylamine, dodecylamine, and dioctadecyldimethylammonium bromide improve cellular uptake and mucosal adhesion, while anionic charge modifiers such as sodium stearate provide repulsion-based stability and controlled drug release. Penetration enhancers as well prodrugs can increase drug permeability across biological barriers like the skin, cornea, mucosal surfaces, and the blood–brain barrier by modifying the lipid bilayer and enhancing membrane fluidity [149,150,151,152,153]. Common penetration enhancers used in SLNs include oleic acid, ethanol, menthol, and linoleic acid. Targeting ligands on SLNs enables site-specific drug delivery by binding to specific receptors on diseased cells, enhancing drug accumulation while minimizing off-target effects [154]. This improves therapeutic efficacy and reduces systemic side effects. Common targeting ligands include folic acid for cancer targeting, transferrin for tumor and brain delivery, aptamers for precise molecular recognition, monoclonal antibodies for immunotherapy, and peptides for receptor-mediated drug delivery. Polymer coatings enhance SLN stability, regulate drug release, and provide stealth properties to evade immune detection, prolonging circulation time. They also improve mucoadhesion and enable site-specific delivery [155,156]. Common polymer coatings include chitosan for mucoadhesion and controlled release, hyaluronic acid for tumor targeting [157], PEG for stealth properties and extended circulation, and polyvinyl alcohol for improved nanoparticle dispersion and stability [158,159]. The strategic selection of these excipients significantly improves SLN-based drug delivery by optimizing stability, bioavailability, and therapeutic efficiency. Typical structural components of SLNS with suitable examples are depicted in Table 3.

### 7.2. Role of SLNs for Anticancer Phytochemical Delivery

SLNs play a pivotal role in enhancing the delivery of anticancer phytochemicals, which typically suffer from low water solubility and limited bioavailability. The lipid matrix shields sensitive phytochemicals from degradation caused by light, oxygen, and heat, thereby enhancing their shelf life and therapeutic effectiveness.

The low oral bioavailability of curcumin can be addressed through absorption enhancers and formulation strategies; however, even with bioenhancers like piperine, the resulting improvement remains insufficient for achieving optimal therapeutic efficacy [26,68]. SLN-formulated curcumin significantly enhanced curcumin’s bioavailability and anticancer efficacy in Hodgkin’s lymphoma [160]. The formulated curcumin-loaded SLNs reduced tumor growth more effectively than free curcumin, suppressed key pro-survival proteins (XIAP and Mcl-1) and inflammatory cytokines (IL-6 and TNF-α), and showed additive effects with chemotherapy. Studies demonstrated that N-trimethyl chitosan-grafted SLNs significantly enhanced brain delivery and bioavailability of compounds like curcumin and resveratrol [161]. These findings highlight the promise of surface-engineered SLNs in facilitating effective CNS-targeted delivery of poorly bioavailable phytochemicals. Modifying the surface of nanomedicines can improve phytochemical delivery and help overcome drug resistance by altering their biophysical interactions with cancer cell membrane lipids, thereby facilitating greater accumulation of phytochemicals in target tissue [162,163].

For instance, N-trimethyl chitosan-palmitic acid-coated SLNs increased resveratrol bioavailability by 3.8 times, and N-carboxymethyl chitosan-coated SLNs improved curcumin uptake in lymphatic cells by 6.3 times, with a 9.5-fold increase in oral bioavailability compared to suspension forms [161,164]. Thymoquinone poses challenges for oral delivery due to its high lipophilicity, low bioavailability, and instability in the gastrointestinal environment. Optimized chitosan-modified thymoquinone-SLNs demonstrated particle sizes ranging from 135 to 211 nm, high drug entrapment efficiency (up to 91.78%), and positive surface charge of +12.52 mV [165]. These nanoparticles exhibited sustained drug release over 24 h and notable mucoadhesive efficiency (~67%). Compared to drug suspension, the SLNs showed enhanced intestinal permeation and significantly improved oral bioavailability in Wistar rats. Chitosan-coated SLNs offer a promising strategy to improve mucosal delivery of bioactives due to their biocompatibility, biodegradability, low toxicity, mucoadhesive properties, antimicrobial effects, and ability to enhance drug absorption. A chitosan-based SLN system was developed to improve the bioavailability of apocynin, a phytochemical with diverse pharmacological potentials including antitumour efficacy but poor pharmacokinetics [166]. The optimized SLN formulation, featuring a chitosan-apocynin core and a polyvinyl alcohol coated shell, demonstrated significantly improved oral and intravenous bioavailability in rats compared to the drug solution.

Lipid-based carriers loaded with drugs or phytochemicals have been shown to exhibit higher cytotoxicity than conventional formulations, primarily due to enhanced cellular uptake via endocytosis. Some studies also report the ability of certain lipid nanoparticles to penetrate cell nuclei, particularly when surface-modified with targeting ligands like folic acid or combined with components like protamine, facilitating nuclear delivery in cancer and fibroblast cells [167,168]. Nuclear accumulation of lipid carriers is beneficial for delivering anticancer agents, but excessive penetration time may limit therapeutic effectiveness. SLNs formulated with paraffin oil and stearic acid, and stabilized by Tween 60 and Span 60, exhibited excellent aggregation stability for over 90 days, with particle sizes ranging from ~30 to 50 nm [169]. Cellular uptake studies in C6 and MCF-7 cancer cells revealed size-dependent internalization, with ~50 nm particles entering cells within 1 h. When loaded with doxorubicin or thymoquinone, these SLNs showed markedly higher cytotoxicity against MCF-7 and HTC 116 cell lines compared to the free drug, while blank SLNs remained largely non-toxic. Their ability to localize in the nucleus supports their promise for anticancer therapy by facilitating DNA replication disruption and triggering apoptosis.

Phytochemicals such as flavonoids, polyphenols, and terpenoids have demonstrated strong anticancer potential due to their high efficacy and low toxicity. They exert their effects by modulating cancer-related processes like apoptosis, pyroptosis, autophagy, migration, and senescence through the regulation of reactive oxygen species, MAPK, NF-κB, DLC1, and glycolytic enzyme pathways [170]. Epigallocatechin gallate, a green tea catechin with known anticancer activity, suffers from poor bioavailability and instability at physiological pH. Encapsulating catechin within SLNs was found to enhance its stability [171]. The developed SLNs significantly improved cytotoxicity showing 8.1-fold and 3.8-fold higher anticancer activity against MDA-MB 231 breast and DU-145 prostate cancer cells, respectively, compared to free drug. Lung cancer involves critical molecular alterations such as DNA damage, epigenetic changes, and mutations in key genes like TP53, KRAS, and EGFR, which activate abnormal signaling pathways that promote tumor growth and metastasis [172,173]. Phyllanthi tannin fraction was encapsulated into SLNs using the thin-film hydration method with Brij^®^58, glyceryl monostearate, and lecithin as formulation components [174]. The tannin-rich extract SLNs exhibited enhanced cytotoxicity and apoptosis induction, with a markedly lower IC_50_ value (20.74 μg/mL) than that of the free fraction (67.43 μg/mL). In vivo studies further confirmed improved therapeutic outcomes, as the SLNs achieved a higher lung tumor inhibition rate of 64.55% at a lower dose (0.4 g/kg) compared to 59.97% at a higher dose (2 g/kg) with the free extract. Curcumin-loaded SLNs significantly enhanced curcumin’s therapeutic efficacy for lung cancer by improving bioavailability, cellular uptake, and tumor targeting after intraperitoneal administration [175]. The nanoparticles (20–80 nm) reduced the IC_50_ in A549 cells to 4 μM and increased in vivo tumor inhibition from 19.5% to 69.3%, mainly through apoptosis, highlighting their potential in lung cancer treatment and anticancer drug development.

Mannose-coated SLNs enhance targeted delivery of anticancer agents by facilitating receptor-mediated uptake via mannose-specific lectin receptors overexpressed on tumor-associated macrophages and dendritic cells within the tumor microenvironment, thereby improving drug accumulation at the tumor site [176].

Mannose-modified SLNs were developed for targeted curcumin delivery in lung disease treatment. Structural and physicochemical analyses confirmed successful formulation, with improved encapsulation efficiency and drug release [177]. In A549 lung carcinoma cells, the prepared SLNs enhanced cellular uptake and cytotoxicity, though free curcumin showed greater effects in inhibiting cell migration and inducing apoptosis. Notably, the developed SLNs demonstrated superior antibacterial activity against *Mycobacterium intracellulare* and infected macrophages. The methoxy phenyl ester derivative of curcumin formulated into SLNs demonstrated high entrapment efficiency (96.8%), uniform particle size (113 nm), low polydispersity index (PDI, 0.177), and adequate drug loading of 6.2% [178]. The optimized formulation provided sustained drug release over 48 h. Pharmacokinetic analysis revealed that the developed SLN significantly extended curcumin’s half-life to 14.7 h, reduced its metabolic rate by 35.6-fold, and increased systemic exposure (AUC_0_-t) by 37-fold compared to free curcumin.

Folate-modified SLNs enable targeted drug delivery by binding to folate receptors overexpressed on cancer cells, thereby enhancing cellular uptake and improving therapeutic efficacy. Folate-modified paclitaxel-loaded SLNs, prepared via nanoprecipitation, showed enhanced targeting of lung tumors by selectively accumulating in folate receptor-expressing cells such as HeLa and M109-HiFR cells [179]. Pulmonary delivery led to improved tumor uptake and significantly increased cytotoxicity, reducing the IC_50_ from 340 to 60 nM, confirming effective receptor-mediated delivery. A comprehensive overview of various phytochemicals with antitumor properties incorporated into SLNs, detailing their preparation techniques, formulation components, and key findings, is summarized in Table 4.

SLNs provide a robust protective matrix for anticancer phytochemicals, preventing rapid degradation and aggregation while enhancing stability and delivery efficiency. When encapsulated in SLNs, sulforaphene maintained stability across a wide pH range (2–9), resisted high-temperature breakdown, and retained integrity during 8 weeks of storage at 25 °C [180]. Furthermore, SLN-loaded sulforaphene demonstrated sustained release, improved intestinal absorption, and comparable anticancer efficacy against A549 lung cancer cells to its free-form counterpart. Similarly, 4-hexylresorcinol (4-HR), a hydrophobic chemoprotective agent, forms aggregates that limit bioavailability. Encapsulation into SLNs (169–645 nm size, 75–96% entrapment efficiency) significantly enhanced cellular uptake and cytotoxicity across multiple cancer cell lines including HeLa, A549, and CT-26 achieving up to a 6.4-fold improvement in anticancer effect compared to the free compound [181].

**Table 4:** Phytochemicals, formulation techniques, components, and key characteristics of SLN-based delivery systems.

Phytochemicals	Preparation Method	Composition	Therapeutic Application	Highlights	Reference
Allicin	High-pressure homogenization	Stearic acid, Tween 80 and lecithin	Lung cancer	The formulated allicin-loaded SLNs measured 67.01 nm in size with a surface charge of −29.29 mV. SLNs significantly reduced the viability of A549 cancer cells after 48 h, while normal HFF cells remained unaffected. Flow cytometry analysis indicated an increase in the subG1 peak and a notable upregulation of caspase-3 and caspase-9, with minimal impact on caspase-8, highlighting an intrinsic apoptotic pathway. Additionally, the SLNs exhibited strong antioxidant activity, effectively inhibiting ABTS and DPPH free radicals.	[182]
Lawsone	Hot homogenization	Precirol^®^, Tween 80, Poloxamer 407	Lung carcinoma	The mean diameters of free SLNs and phyto-constituent SLNs were 97 ± 1.4 nm and 127 ± 3.1 nm, respectively. Developed SLNs exhibited high encapsulation efficiency (95.88 ± 3.29%) and drug loading capacity of 22.72 ± 1.39 mg/mL. Cytotoxicity assays showed that plain lawsone inhibited A549 cell growth with IC_50_ values of 17.99 ± 1.11, 13.37 ± 1.22, and 9.21 ± 1.15 μg/mL at 24, 48, and 72 h, respectively. The SLNs exhibited stronger cytotoxic effects after 48 h (IC_50_: 9.81 ± 1.3 μg/mL). SLNs (13.37 ± 1.22 μg/mL) induced ~52% apoptosis and necrosis after 48 h. qPCR results indicated Bcl-2 downregulation and caspase-9 upregulation, confirming apoptosis induction in A549 cells.	[183]
Lycopene	Hot homogenization and ultrasonication	Stearic acid, Poloxamer 407, Soy phosphatidylcholine/Soy lecithin.	Antioxidant and Anti-melanogenic	The particle size analysis of lycopene-loaded SLNs revealed an average size of 151.1 ± 2.3 nm. Electron microscopy examination confirmed that the nanoparticles were spherical, with an encapsulation efficiency of 85.76 ± 2.75%. Assessment of the anti-tyrosinase effects of SLNs demonstrated a significant reduction in cellular tyrosinase activity, melanin synthesis, and reactive oxygen species levels. Notably, SLNs effectively inhibited melanin production while exhibiting minimal toxicity toward melanoma cells.	[184]
*Morus alba* extract	High-pressure homogenization	--	Cytotoxicity	The optimized SLNs exhibited favorable physicochemical properties and significantly enhanced cytotoxicity and apoptosis compared to extract alone (*p* < 0.05) using MDA-MB231 cell line. They effectively disrupted DNA replication and cell division by inhibiting the S (9.7 ± 1.7%) and G2/M (2.2 ± 0.6%) phases. The apoptosis rate was notably higher (*p* < 0.05 in SLNs (81.46%) than in extract alone (72.49%), confirming their superior therapeutic potential.	[185]
Thymoquinone	Oil-in-water microemulsion	--	Brain malignancies	Thymoquinone-encapsulated Eudragit L100-coated SLNs released the highest drug content (78.215 ± 0.749%) at pH 5.5 after 22 h. Pharmacokinetic and biodistribution studies indicated that, 48 h post-administration, drug accumulated in various organs, including the brain (16.5 ± 1.5%), kidneys (21.167 ± 1.041%), heart (12.125 ± 0.781%), liver (16.375 ± 1.317%), lungs (13.5 ± 1.8%), and another unspecified tissue (17.15 ± 1.5%). Molecular modeling demonstrated that thymoquinone exhibited strong binding affinity to EGFR (−7.8 kcal/mol), comparable to the reference drug temozolomide.	[186]
Curcumin	High shear homogenization	Cholesterol, Poloxamer-188	Breast cancer	The optimized formulation (Chol-CUR SLN) exhibited a uniform particle size of 166.4 ± 3.5 nm and a high encapsulation efficiency of 76.9 ± 1.9%. In vitro experiments on MDA-MB-231 human breast cancer cells demonstrated enhanced cellular uptake and significantly greater cytotoxicity for Chol-CUR SLNs compared to free curcumin. Additionally, exhibited markedly higher levels of apoptosis, indicating its improved therapeutic potential.	[187]

### 7.3. Co-Loaded Phytochemicals in SLNs

SLNs co-encapsulating multiple phytochemicals or a phytochemical–drug combination represent a promising approach to boost the therapeutic efficacy of plant-based bioactives. Co-loading within a single SLN system can result in synergistic effects by targeting multiple pathways simultaneously, particularly in diseases such as cancer, inflammation, and microbial infections [188]. Moreover, codelivery of phytochemicals alongside chemotherapeutic agents has been shown to counteract chemoresistance by enhancing drug uptake in cancer cells, suppressing drug efflux transporters, and inhibiting DNA repair and resistance protein expression [43]. Conventional chemotherapy often requires high doses to obtain therapeutic effects, which can lead to serious side effects such as cardiotoxicity, nephrotoxicity, ototoxicity, and hepatotoxicity [189,190,191]. Incorporating antioxidants through co-delivery strategies has also demonstrated the potential to reduce toxicity and minimize side effects through the modulation of various biological pathways, thereby enabling better treatment adherence and improved outcomes in terms of tumor response and patient survival [192]. For example, biotin/lactobionic acid-modified PEG–PLGA–PEG nanoparticles co-loaded with curcumin and 5-fluorouracil showed enhanced cellular uptake, increased intracellular delivery, and superior cytotoxicity against tumor cells, resulting in improved synergistic anticancer efficacy in hepatocellular carcinoma [193]. Chitosan-coated SLNs co-loaded with trans-resveratrol and ferulic acid, and conjugated with folic acid, demonstrated promising colon targeting efficacy [194]. In vitro anticancer studies using HT-29 colon cancer cells showed significantly enhanced cytotoxicity and apoptosis induction compared to the free drug combination. SLNs co-loaded with curcumin and paclitaxel showed enhanced pharmacokinetic parameters, including increased area under the curve, prolonged drug residence time, and extended half-life, contributing to longer systemic circulation [195]. Notably, the lung tumor suppression rate achieved with the combined SLN formulation was 78.42%, compared to 40.53% with paclitaxel alone and 51.56% with the non-nanoformulated drug combination.

**Figure 3:**
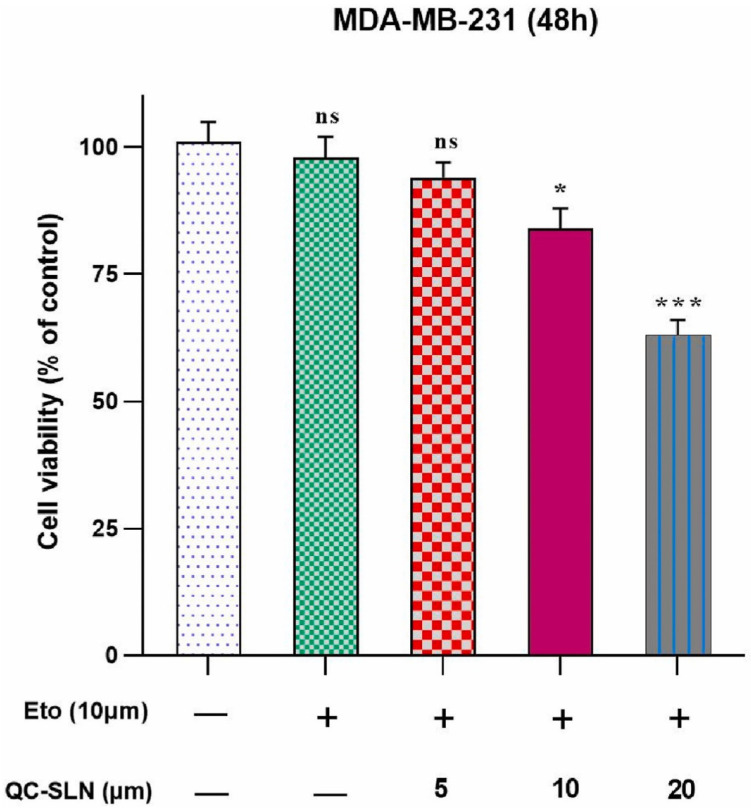
Cell Viability determination through MTT Assay in MDA-MB-231 cells. Reproduced from Ref. [197]. ns: not significant, * *p* < 0.05 and *** *p* < 0.001, as compared to control.

A study explored the cardioprotective effects of berberine-loaded SLNs against doxorubicin-induced cardiotoxicity in vitro [196]. SLNs were formulated using the microemulsion method with tripalmitin, Tween 80, and poloxamer 407, and exhibited favorable characteristics, including a small particle size (13.12 nm), 50% entrapment efficiency, and good stability. In H9c2 cardiomyocytes, prepared SLNs significantly enhanced cell viability and reduced doxorubicin-induced cytotoxicity, oxidative stress, and apoptosis, showing comparable or superior effects to free berberine. These findings suggest that SLNs represent a promising and cost-effective delivery approach for mitigating chemotherapy-related cardiac damage. Quercetin-loaded SLNs, when administered in combination with etoposide, significantly enhanced the inhibition of MDA-MB-231 breast cancer cell proliferation compared to treatment with etoposide or quercetin SLNs alone [197]. This combined therapy also strongly promoted apoptosis, as evidenced by an increased Bax/Bcl-2 gene ratio, elevated expression of p53 and p21 proteins, and activation of caspase-3 and -9. These findings highlight the potential of the combination as an effective therapeutic approach for breast cancer, particularly in overcoming resistance to etoposide (Figure 3). Thus, in general, nano co-delivery for cancer treatment faces clinical challenges related to safety, drug interactions, and nanomaterial biosafety. However, combining plant-based compounds with chemotherapeutics via targeted nano-delivery systems offers a promising strategy, with the potential for enhanced efficacy and reduced toxicity in future clinical applications.

## 8. Nanostructured Lipid Carriers (NLCs)

NLCs are a drug delivery system comprising unstructured colloidal lipid particles (≤100 nm) formed by combining solid and liquid lipids and dispersing them in an aqueous phase with the aid of emulsifying agents. Solid and liquid lipids are typically mixed in ratios ranging from 70:30 to 99.9:0.1, with surfactant concentrations generally falling between 1.5% and 5% (*w*/*v*) [198]. This structure creates an amorphous lipid matrix with improved polymorphic behavior, reducing drug expulsion and enhancing formulation performance [199]. One proposed mechanism suggests that the embedded fluid lipid generates nanoglobules within the solid lipid matrix, thereby improving drug solubilization and enhancing formulation stability. NLCs are also classified into three types based on their structural characteristics and lipid composition: imperfect crystal type (Type I), amorphous type (Type II), and multiple type (Type III) (Figure 2). The imperfect crystal type is formed by mixing lipids of varying chain lengths, creating structural voids that enhance drug entrapment. The amorphous type incorporates medium-chain triglycerides with solid lipids, preventing recrystallization and improving storage stability. The multiple type consists of solid lipids blended with oils, forming nanocompartments that significantly increase drug loading capacity [200,201]. These structural modifications result in a less ordered lipid matrix by reducing lipid crystallinity. Compared to SLNs, this results in increased drug entrapment efficiency, enhanced retention, and improved long-term stability by minimizing drug expulsion during storage, making NLCs highly effective for drug delivery applications [115]. Lipid nanoparticles like NLCs address several common formulation challenges linked to polymeric nanoparticles, including cytotoxicity, reliance on organic solvents, and obstacles in scaling up for commercial manufacturing [112]. Studies suggest that SLNs exhibit a slower drug release rate than NLCs at low drug encapsulation levels, though this difference diminishes with higher drug loading. Additionally, NLCs have demonstrated greater stability than SLNs when stored at room temperature. Despite their lower water content compared to conventional emulsions, NLC formulations still require preservation to prevent microbial contamination and maintain nanoparticle stability, including particle size. This can be achieved either by freeze-drying (lyophilization), which removes water and converts the suspension into a porous solid form, or by incorporating suitable preservatives to retain stability in the liquid state [202]. It was reported that trehalose, as a cryoprotectant, effectively prevented NLC aggregation across all tested concentrations (5, 10, and 15% *w*/*v*). Recently, a study applied Neurofuzzy Logic to optimize the lyophilization of NLCs and evaluate carbohydrate cryoprotectants. The technique identified the molecular weight of cryoprotectants as a key factor in freezing conditions and concentrations, leading to a traffic light system for selecting optimal sugars for NLC stabilization [203].

NLCs enhance oral active absorption by promoting uptake through intestinal M-cells and bypassing first-pass hepatic metabolism. They facilitate transport via transcellular and paracellular routes while inhibiting P-glycoprotein and cytochrome P450 enzymes. The lipid components of NLCs also trigger chylomicron formation, enabling lymphatic drug transport [204]. Upon oral administration, NLC lipids undergo enzymatic digestion in the stomach and small intestine, producing monoglycerides and free fatty acids. Their nanoscale size increases surface area, prolongs gastrointestinal residence time, and improves interaction with the mucosal surface, thereby enhancing absorption [205]. Positively charged coatings (e.g., chitosan, benzalkonium chloride) enhance mucoadhesion by binding to the negatively charged intestinal lining, improving drug uptake [206]. NLCs functionalized with a polycationic cell-penetrating peptide and coated with polyphosphates enabled enzyme-triggered charge conversion at target cells by intestinal alkaline phosphatase [207]. Surfactants like poloxamer increase intestinal permeability by opening tight junctions and inhibiting P-glycoprotein efflux, supporting paracellular and intracellular drug transport [208]. Additionally, lipid digestion stimulates bile secretion, leading to the formation of mixed micelles that aid drug transfer across the unstirred water layer to enterocytes [209]. These micelles are selectively absorbed into the lymphatic system, effectively bypassing hepatic metabolism, which enhances the bioavailability of highly metabolized drugs, reduces dosing frequency, and minimizes side effects [210]. NLCs enhance flavonoid delivery by improving their absorption, solubility, and bioavailability, leading to greater therapeutic efficacy. Additional advantages include improved permeability, extended half-life, minimized side effects, and targeted systemic delivery [211,212].

NLCs offer an effective approach to improve the stability of anticancer phytochemicals such as curcumin, which is notoriously prone to photodegradation and instability under physiological conditions [213]. In a recent study, NLCs encapsulating curcumin (particle size ~154 nm, PDI ~0.25, entrapment efficiency > 95%) significantly protected it from light-induced degradation. Specifically, the photostability of curcumin increased from approximately 9.6 h to 19.3 h, representing nearly a 600% improvement compared to free curcumin formulations. NLCs improve curcumin stability by shielding it from environmental stressors, converting it into an amorphous form to reduce degradation, and incorporating antioxidants and light-absorbing oils, which together significantly lower its degradation rate.

Despite several benefits, NLCs have certain limitations, including potential lipid oxidation, variability in large-scale production, and possible burst drug release [214]. Stability concerns related to lipid crystallization and polymorphic transitions can affect drug loading and release kinetics [215]. Furthermore, regulatory challenges and high manufacturing costs may limit their widespread clinical application. Nonetheless, ongoing advancements in formulation strategies and lipid nanotechnology continue to optimize NLCs for efficient phytochemical delivery, making them promising carriers in nutraceutical and pharmaceutical fields.

A diverse range of lipids is used in the formulation of NLCs to improve the oral bioavailability of poorly soluble drugs through different mechanisms. The selection of suitable lipids depends on factors like physiological compatibility, physicochemical characteristics, drug solubility, and miscibility between solid and liquid lipids. For instance, it has been reported that Miglyol is incompatible with several solid lipids such as Suppocire A, Geleol, Cacao Butter, and Witepsol E75, but shows compatibility with Compritol 888 ATO and Gelucire 43/01 [216]. Ideal lipids should be GRAS-certified, stable, and able to solubilize or associate with the drug effectively. Structured edible lipids can further improve membrane permeability and drug solubility. These lipids have been reported as promising materials for fabricating NLCs that enhance membrane permeability and improve drug solubility [217]. The typical structural composition of NLCs, along with suitable examples, is presented in Table 5.

### 8.1. Role of NLCs for Anticancer Phytochemical Delivery

In phytochemical delivery, NLCs offer several advantages, including enhanced solubility, improved bioavailability, prolonged systemic circulation, and targeted delivery. Many anticancer phytochemicals, such as curcumin, resveratrol, and quercetin [218,219,220] suffer from poor aqueous solubility, rapid metabolism, and instability under physiological conditions. Encapsulating these bioactive compounds in NLCs helps protect them from degradation, increases gastrointestinal absorption, and facilitates controlled release, ensuring sustained therapeutic effects. Several studies highlight the efficacy of NLCs as efficient vehicles for sustained and targeted delivery of phytochemicals in cancer therapy.

NLCs loaded with quercetin, known for its anticancer activity, along with the bioenhancer piperine, exhibited favorable physicochemical properties, including particle sizes under 180 nm, a polydispersity index below 0.3, and drug entrapment efficiency exceeding 85% [221]. In vitro studies using FaDu oral cancer cells demonstrated enhanced cellular uptake, increased cytotoxicity, and greater mitochondrial membrane depolarization with the dual drug-loaded NLCs compared to the free drugs. Apoptotic activity was further confirmed through flow cytometry. In vivo biodistribution studies using Coumarin-6-labeled NLCs indicated efficient targeting of oral tissues following oral administration. It was reported that isoliquiritigenin-loaded NLCs improved tumor inhibition and achieved a 2.5-fold higher drug concentration at the tumor site in liver cancer-bearing mice compared to free flavonoid [222]. Similarly, NLCs encapsulating zerumbone for leukemia treatment demonstrated sustained drug release following zero-order kinetics and significantly greater cytotoxicity against Jurkat T-cell leukemia cells than free zerumbone [223].

Surface-functionalized NLCs with tumor-homing ligands have the potential to improve the therapeutic efficacy of phytochemical-based cancer treatments. In a recent study, NLCs were loaded with osthole, a coumarin derivative, and surface-modified with chitosan conjugated to folate to target folate receptors, which are overexpressed in many cancer cells [224]. The resulting CS–FA–NLC–Osthole particles had an mean diameter of approximately 179 nm, a positive zeta potential (+19 mV), and a high encapsulation efficiency (~83%). In vitro evaluation employing HT-29 colon cancer cells demonstrated significantly enhanced cellular uptake, selective cytotoxicity, increased expression of apoptosis markers, and superior antioxidant activity compared to free osthole.

Auraptene, a citrus-derived coumarin, was encapsulated within NLCs and coated with chitosan conjugated to folic acid to target folate receptors on ovarian cancer cells (A2780 line). The resulting FA–CS–NLC–Auraptene system featured ~211 nm particles and demonstrated selective cytotoxicity by inducing apoptosis through the upregulation of Bak, Bax, and p53, while sparing normal fibroblasts. This clearly illustrates how surface-functionalized NLCs can enhance tumor selectivity and the therapeutic efficacy of phytochemical-based treatments.

NLCs encapsulating poorly water-soluble bioactives like curcumin have demonstrated notable formulation advantages, including enhanced drug entrapment and loading efficiency, improved release characteristics, and increased stability under both in vitro and in vivo conditions [225]. They also significantly improved solubility and bioavailability, exhibited strong physical stability, and followed a sustained release profile consistent with the Higuchi kinetic model.

Quercetin’s clinical application is limited by poor water solubility, low oral bioavailability, chemical instability, and rapid metabolism. Biocompatible and biodegradable quercetin-NLCs have been fabricated utilizing the phase inversion method, resulting in a formulation with enhanced physical stability compared to native quercetin [226]. In vitro studies revealed that nanoencapsulation increased quercetin’s stability to nearly 95% and that NLCs induced dose-dependent cytotoxicity and apoptosis in MCF-7 and MDA-MB-231 breast cancer cells. Notably, this nanoformulation offers more than three times the efficacy of standard quercetin in reducing cancer cell viability by triggering cell cycle arrest and apoptosis, suggesting it could be a promising breakthrough for breast cancer treatment with minimal side effects.

Phytochemical-loaded NLCs enhance photodynamic cancer therapy by improving drug stability, solubility, and targeted delivery. Upon light activation, these systems generate reactive oxygen species that trigger cancer cell death. The NLCs ensure better tumor accumulation and sustained release, resulting in superior anticancer efficacy compared to free phytochemicals [227]. In summary, NLCs have emerged as a promising platform for the efficient delivery of phytochemicals, addressing key challenges such as poor solubility, low bioavailability, and chemical instability. Table 6 provides a comprehensive summary of various phytochemicals incorporated into NLCs, highlighting their preparation methods, formulation components, and key findings.

### 8.2. Co-Loaded Phytochemicals in NLCs

Co-loading a phytochemical with another compound into NLCs enhances efficacy by enabling synergistic effects, targeting multiple pathways, and overcoming drug resistance. The superior drug loading capacity, improved stability, and minimized risk of drug expulsion typically associated with NLCs further improve therapeutic outcomes.

Although phytochemicals exhibit potent antitumor activity, their clinical application is limited by challenges such as poor bioavailability, limited cellular uptake, low water solubility, rapid distribution to normal tissues, extensive hepatic metabolism, and a narrow therapeutic window [232,233]. Recent research increasingly focuses on combining phytochemicals with chemotherapeutic agents to achieve synergistic therapeutic effects, improve pharmacokinetics, overcome multidrug resistance, and sensitize cancer cells to chemotherapy [234]. A co-loaded NLC system containing raloxifene and naringin was developed to enhance oral delivery for breast cancer treatment [235]. The optimized formulation showed favorable physicochemical properties, high entrapment efficiency, and significantly improved drug release and intestinal permeability compared to drug suspensions. Confocal imaging confirmed deeper tissue penetration, while in vitro DPPH antioxidant assay demonstrated enhanced antioxidant activity for the combination compared to individual components, attributed to their synergistic antioxidant effect. NLCs composed of glyceryl monostearate, medium-chain triglycerides, and poloxamer 188 were fabricated employing the microemulsion method to co-deliver curcumin and temozolomide. This dual-drug formulation targets brain tumors and other cancers by harnessing the synergistic anticancer properties of both agents, curcumin as a natural anti-inflammatory and chemosensitizer, and temozolomide as a standard chemotherapeutic [236].

Co-loading a phytochemical with another compound into NLCs provides therapeutic and formulation benefits by enabling synergistic effects through multi-pathway targeting. For instance, an optimized NLC formulation co-loaded with quercetin and morin demonstrated enhanced cytotoxicity in MCF-7 human breast cancer cell lines, outperforming both the individual compounds and their combined solution form [237]. These results indicate that such a co-loaded NLC system holds significant potential for overcoming chemotherapy resistance in breast cancer therapy. Current research is exploring innovative strategies that combine drug repurposing, phytotherapeutics, and nanodrug delivery to improve treatment effectiveness, minimize toxicity, and address resistance in cancer therapy. Drug repurposing involves identifying new therapeutic uses for existing drugs, offering a cost-effective and time-efficient strategy for cancer treatment [238]. This approach bypasses early-stage drug development hurdles by utilizing agents with well-established safety profiles. A notable example is ivermectin (Ivn), traditionally used as an antiparasitic agent, which has shown significant anticancer activity through mechanisms such as inducing apoptosis, inhibiting cell proliferation, and modulating key signaling pathways.

A novel NLC system was developed to co-deliver Ivn and methyl-dihydrojasmonate (MJ), a phytochemical with antileukemic potential [239]. The resulting Ivn@MJ-NLCs demonstrated desirable physicochemical properties, including a particle size of ~97 nm, low PDI (0.33), and high drug entrapment efficiency (~97.5% for Ivn and ~99.5% for MJ). The formulation enabled sustained drug release, with 83% of Ivn released over 140 h and 81% of MJ over 48 h. In vitro studies on K562 leukemia cells confirmed significant synergistic cytotoxicity (IC_50_ = 35 µg/mL; combination index = 0.59), particularly at low Ivn doses, while retaining good cytocompatibility with oral epithelial cells. Apoptotic activity was supported by enhanced nuclear fragmentation and upregulation of caspase-3 and BAX. Further ex vivo and in vivo assessments validated the formulation’s safety, demonstrating hemocompatibility and organ-level biocompatibility with no harmful effects on blood parameters or liver and kidney tissues.

**Figure 4:**
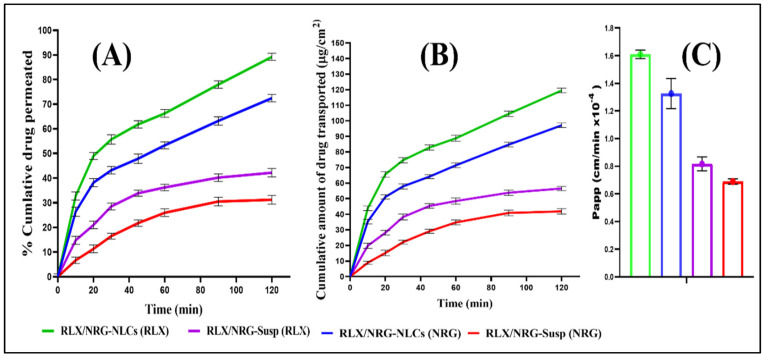
Ex vivo permeation study showing: (**A**) cumulative drug permeation over time, (**B**) cumulative drug transport (μg/cm^2^) over time, and (**C**) apparent permeability coefficients (Papp) of RLX and NRG from the plain suspension and the NLC formulation. Reproduced from Ref. [235].

Breast cancer remains a leading cause of mortality among women globally, with estrogen receptor-positive (ER+) breast cancer being the most prevalent subtype [240]. Its progression is driven by estrogen, which promotes cell cycle progression, survival, and angiogenesis by regulating key proteins such as cyclin D1, Bcl-2, Myc, and VEGF. Raloxifene, initially approved for treating postmenopausal osteoporosis, was later recommended by the FDA as a chemopreventive agent to reduce the risk of invasive breast cancer in this population [241]. A NLC system was developed for the oral co-delivery of raloxifene, a synthetic selective estrogen receptor modulator, and naringin, a flavonoid phytochemical with known anticancer activity, targeting estrogen receptor–positive breast cancer [235]. The optimized RLX/NRG-NLCs exhibited a nanosize of approximately 137 nm and high entrapment efficiencies (~91% for raloxifene and ~85% for naringin). As shown in Figure 4A, RLX and NRG exhibited significantly higher cumulative intestinal permeation from NLCs (89.18% and 72.45%, respectively) compared to their suspensions (42.16% and 31.2%), reflecting a 2.3- and 2.1-fold improvement. Figure 4B confirms this enhanced transport through increased permeation flux from the NLCs. In Figure 4C, the apparent permeability coefficients (Papp) for RLX and NRG from NLCs were nearly 2-fold higher than those from suspensions, indicating superior intestinal absorption potential of the NLC formulation.

The authors suggested that the enhanced intestinal permeability of NLCs is attributed to their nanoscale size, improved drug dissolution, and the inclusion of lipids such as Compritol 888 ATO and oleic acid, which facilitate mucosal transport. Additionally, surfactants like Tween 80 and Labrasol improve drug solubility, enhance membrane penetration, and may inhibit P-glycoprotein (P-gp) efflux, thereby collectively enhancing drug absorption and oral bioavailability. Additionally, the formulation showed improved antioxidant activity and a favorable safety profile in acute toxicity studies in rats, supporting its potential as a safe and effective oral strategy for breast cancer therapy.

Co-delivery using inhalable lipid-based nanoformulations from natural products offers a targeted and effective strategy for pulmonary diseases by promoting bioavailability, minimizing adverse effects, and improving treatment outcomes, especially in chronic respiratory conditions. A co-delivery system of doxorubicin and paclitaxel was developed using emulsion solvent evaporation and ultrasonication methods, incorporating soya lecithin, oleic acid, and Cremophor EL [242]. The resulting dry powder inhalers had an aerodynamic diameter of 394.1 ± 5.6 nm and were intended for lung cancer treatment. Organ distribution studies showed that dry powder containing Cremophor EL facilitated enhanced lung drug deposition compared to plain drugs and other formulations. Additionally, treated animals exhibited no tissue damage and maintained normal behavior 24 h post-treatment. Overall, the co-delivery of anticancer phytochemicals using NLCs presents a promising strategy to enhance therapeutic efficacy, overcome biological barriers, and reduce dose-related toxicity.

## 9. Preparation Methods

The formulation methods of SLNs and NLCs are largely similar, but the key difference lies in the lipid composition, which influences the final nanocarrier structure and performance [123,243]. The preparation techniques primarily rely on high-energy, low-energy, or organic solvent-based methods. High-energy methods, such as high-pressure homogenization and ultrasonication, use mechanical forces to break down lipid droplets into nanoparticles. The high-pressure method, which includes hot and cold homogenization, is widely used due to its scalability and ability to produce stable nanoparticles. In hot homogenization, lipid and drug are melted together, then dispersed in an aqueous phase and homogenized at high pressure, whereas cold homogenization involves solidifying the drug-lipid mixture before homogenization to minimize drug degradation. Low-energy techniques, like microemulsion and phase inversion methods, take advantage of temperature or compositional changes to facilitate nanoparticle formation. Organic solvent-based approaches, such as the emulsification-solvent evaporation technique, where lipids and drugs are dissolved in organic solvents, emulsified in an aqueous phase, and then evaporated to form SLNs. The solvent diffusion method allows for controlled precipitation of lipid nanoparticles by diffusing a water-miscible solvent into an aqueous phase. Microemulsion-based techniques involve the formation of thermodynamically stable oil-in-water microemulsions, which are then cooled to precipitate SLNs. Additionally, techniques like ultrasonication, spray drying, and supercritical fluid processing have been explored for SLN production, each offering unique advantages in particle size control, drug loading, and stability The selection of the method is influenced by factors like the physicochemical characteristics of the phytochemical and lipid, the target particle size, and the stability requirements of the nanocarriers. Table 7 presents a detailed comparison of the widely used preparation techniques for SLNs and NLCs, outlining the corresponding processes, mechanisms, advantages, limitations, and key pharmaceutical applications.

## 10. In Vitro Characterization Techniques

In vitro characterization of SLNs and NLCs is essential for evaluating their stability, drug-loading efficiency, release profile, and biocompatibility. Key assessments include particle size, PDI, and zeta potential, measured via dynamic light scattering to determine uniformity and stability. Morphological analysis using transmission electron microscopy and scanning electron microscopy reveals SLN shape and surface properties. Encapsulation efficiency and drug loading are quantified via high-performance liquid chromatography to assess drug retention. Lipid characterization in SLNs is essential to prevent unwanted crystallization, polymorphic transitions, and stability issues. Differential scanning calorimetric technique and X-ray diffraction method examine lipid crystallinity and drug dispersion, while Fourier transform infrared spectroscopy detects potential drug-lipid interactions. Additionally, techniques such as polarized light microscopy, nuclear magnetic resonance, and thermogravimetric analysis provide valuable insights into lipid behavior during formulation and storage. These analyses ensure optimized drug loading, controlled release, and improved stability, distinguishing SLNs from other lipid-based nanoparticles. In vitro drug release studies using the dialysis bag method or Franz diffusion cell provide insight into release kinetics, modeled using zero-order, first-order, Higuchi, or Korsmeyer-Peppas equations. Stability studies under ICH guidelines monitor particle size, zeta potential, and drug content over time. Additionally, hemocompatibility (haemolysis assay) and cytotoxicity (MTT assay) assess SLN safety for biological applications, and permeation studies evaluate drug transport across biological membranes. These tests collectively ensure that SLN formulations are optimized for therapeutic use. Table 8 provides an overview of the key in vitro and preclinical evaluation techniques employed in the development of phytopharmaceutical formulations.

## 11. Clinical Trials, Patents and Regulatory Aspects

Currently, the clinical translation of anticancer phytochemicals formulated in SLNs and NLCs remains limited, with most investigations restricted to the preclinical stage. Among the compounds explored, curcumin stands out as the only phytochemical to have reached a registered clinical trial (NCT02439385), evaluating nanostructured lipid curcumin particles in combination with FOLFIRI and bevacizumab in metastatic colorectal cancer. For other anticancer phytochemicals, no clinical studies with NCT registration currently exist in SLN/NLC formats, indicating that clinical development is still in its early stages, with curcumin serving as the leading candidate.

Recent patent filings reflect growing interest in leveraging SLNs and NLCs for the targeted and sustained delivery of anticancer phytochemicals. These lipid-based systems aim to overcome common limitations of phytochemicals, such as poor solubility, low bioavailability, and rapid degradation. In the case of SLNs, several phytochemicals with antitumor like silymarin, quercetin, hesperidin, curcumin, indirubin, berbamine, resveratrol, and naringenin, have been successfully encapsulated. For example, folic acid-conjugated silymarin SLNs were developed for lung tumor targeting, while co-loaded SLNs containing quercetin and microRNA-150 improved dual delivery efficiency. Hesperidin and naringenin SLNs enhanced oral bioavailability significantly, with naringenin SLNs showing a 3.1-fold increase in bioavailability in vivo.In parallel, NLCs have enabled further improvements in loading capacity and drug stability. Patented NLC formulations include resveratrol, curcumin, temozolomide-curcumin, and quercetin, among others. Notably, transferrin-conjugated NLCs co-loaded with docetaxel and quercetin were designed for glioblastoma therapy, showing enhanced brain targeting and synergistic cytotoxicity. Similarly, quercetin-loaded NLCs for breast cancer demonstrated extended circulation, cellular targeting, and reduced systemic toxicity. Together, these patents underscore the therapeutic value of SLNs and NLCs in cancer nanomedicine, especially for phytochemicals with promising bioactivity but limited pharmacokinetics. They also emphasize innovations in targeting ligands, dual drug loading, and administration routes such as oral and intranasal delivery for site-specific action. A summary of recent patent filings on SLNs and NLCs, highlighting key innovations, is presented in Table 9 and Table 10, respectively.

Regulatory aspects are central to the successful development and approval of lipid-based nanocarriers for anticancer phytochemicals, as these systems are governed within the broader category of nanomedicines. Both the U.S. FDA and the European Medicines Agency (EMA) mandate rigorous characterization beyond conventional formulations, including detailed assessment of particle size distribution, surface charge, morphology, stability, and release kinetics. The EMA reflection paper on nanotechnology-based medicinal products for human use outlines regulatory expectations for nanocarriers, emphasizing critical quality attributes, toxicological evaluation, and comparability studies in the event of manufacturing changes. Similarly, the FDA promotes a Quality by Design (QbD) approach, in alignment with ICH Q8–Q10 guidelines, which emphasize systematic pharmaceutical development, risk management, and lifecycle monitoring [277]. Case studies of approved lipid-based nanomedicines, such as Doxil^®^ (liposomal doxorubicin) and Onpattro^®^ (patisiran, a lipid nanoparticle-based siRNA therapy), demonstrate how lipid carriers can advance through clinical evaluation with comprehensive toxicological and pharmacokinetic profiling [278]. For phytochemicals, additional hurdles include raw material standardization, reproducibility of formulation, and robust evidence of improved pharmacokinetics and therapeutic efficacy. Incorporating regulatory guidance and leveraging lessons from existing lipid-based nanomedicines can therefore provide a clear roadmap for translating phytochemical-loaded SLNs and NLCs from preclinical promise to clinical approval.

## 12. Advancements, Challenges, and Future Directions

The use of lipid nanoparticles for phytochemical delivery holds significant promise for improving the therapeutic efficacy and bioavailability of natural compounds in the future. These carriers enable targeted drug delivery, enhance solubility, and prolong systemic circulation, thereby overcoming the limitations of traditional formulations. Developing a stable and effective delivery system for natural compounds requires a thorough understanding of their physicochemical properties, compatibility with phytochemicals and excipients, suitable formulation and characterization techniques, and ideal storage conditions under various environmental stresses [279]. Stimuli-responsive SLNs and NLCs have advanced the targeted delivery of anticancer phytochemicals by enabling controlled drug release in tumor environments. pH-sensitive systems with quercetin show improved efficacy and reduced toxicity in lung cancer [280]. However, challenges like scalability and tumor heterogeneity remain, highlighting the need for biodegradable, multi-responsive carriers for clinical use [98].

Hybrid lipid–polymer nanoparticles are advanced nanocarrier systems that integrate the structural and functional advantages of both polymeric nanoparticles and lipid-based carriers. Typically composed of a biodegradable polymeric core (e.g., polycaprolactone or PLGA) and a lipid shell (e.g., phospholipids, cholesterol, or cationic lipids), these systems provide enhanced mechanical stability, controlled drug release, and biocompatibility [281]. The lipid layer improves cellular uptake and bioavailability, while the polymeric matrix ensures sustained drug release and protection of encapsulated agents. This hybrid nanoparticle composed of polycaprolactone, lycopene, ethylene glycol, and didodecyldimethylammonium bromide was formulated via bulk nanoprecipitation to co-deliver insulin-like growth factor receptor 1 siRNA and the anticancer phytochemical, lycopene [282]. The formulation demonstrated significant in vitro inhibition of MCF-7 breast cancer cell proliferation by inducing apoptosis, attributed to the synergistic effects of gene silencing and the antioxidant-mediated anticancer activity of lycopene.

Efficient extraction and processing techniques such as supercritical fluid extraction are crucial in ensuring the microbial safety and quality of herbs and spices, as conventional methods may inadvertently retain pathogens along with the bioactive constituents [283]. Continued innovation in extraction, purification, and separation technologies is essential to improve the efficiency, safety, and cost-effectiveness of herbal processing, ensuring the delivery of high-quality phytopharmaceuticals with minimal microbial load and maximal therapeutic potential. The processes involved in collecting, isolating, purifying, and grading nutraceuticals can lead to degradation, chemical instability, and a reduction in the quality of the active constituents due to exposure to heat, light, oxygen, and mechanical stress [284]. Contamination and both intentional and unintentional adulteration are key challenges that affect the quality recovery of products from natural sources. Quantifying a phytochemical can be difficult when validated analytical and bioanalytical characterization methods are lacking. In addition to other analytical challenges, major concerns involve selecting suitable isolation and sampling techniques, the absence of standardized regulatory protocols, and the lack of dependable reference material [285]. Nanoparticles sized between 100 and 1000 nm are likely to undergo phagocytosis, whereas those less than 100 nm are susceptiblle to endocytosis, requiring further screening to safeguard healthy cells and ensure therapeutic safety. While phospholipid-based nanoformulations are generally regarded as biocompatible, comprehensive studies are still necessary to fully establish their safety profile [286].

Nanoformulations exhibit size-dependent distribution and are highly sensitive to formulation changes, making large-scale production challenging and often resulting in reduced reproducibility and variable therapeutic outcomes [287]. Owing to the potential for significant variability and low reproducibility, employing a QbD and systematic evaluation strategy is essential for controlling key physicochemical variations during formulation and ensuring uniformity across production batches [288]. Despite these advancements, challenges remain, including scale-up, storage stability, and regulatory approval of complex nanostructures. Variability in encapsulation efficiency and high production costs also limit clinical translation. Strategies like composition optimization, surface modification, and stimuli-responsive design are being explored to overcome these limitations. Future research should prioritize the development of scalable, cost-efficient processes and stable, biomimetic nanostructures to improve delivery efficiency. Interdisciplinary collaboration and regulatory alignment are essential to realizing the full clinical value of phytochemicals through these advanced delivery systems.

## 13. Conclusions

SLNs and NLCs have demonstrated significant potential as effective nanocarriers for anticancer phytochemical delivery, successfully addressing challenges such as instability, poor bioavailability, and limited solubility. This review provides an overview of various developments in formulation strategies and co-delivery approaches to maximize their therapeutic potential. Although SLNs give stability and biocompatibility, NLCs offer better drug loading and storage capabilities. Future research on phytochemical-loaded SLNs and NLCs should progress beyond small Phase I/II studies toward multicenter Phase II/III clinical trials that can rigorously validate safety, optimize dosing strategies, and confirm efficacy across diverse cancer types. From a regulatory perspective, stronger alignment with FDA and EMA frameworks, informed by lessons from approved nanomedicines, will be crucial for minimizing developmental risks and facilitating smoother approval pathways. On the technological side, priority should be given to the development of advanced systems, including ligand-decorated, hybrid lipid–polymer nanoparticles, and stimuli-responsive lipid nanocarriers, along with the establishment of scalable, GMP-compliant manufacturing platforms supported by robust QbD principles. Therapeutically, integrating phytochemical nanocarriers with conventional chemotherapy or immunotherapy holds promise for synergistic benefits, potentially minimizing toxicity and overcoming drug resistance. Overall, a coordinated focus on translational research, regulatory harmonization, and clinical validation will be critical to unlocking the full therapeutic potential of SLNs and NLCs in oncology.

## Figures and Tables

**Table 1:** Summary of key anticancer phytochemicals, their botanical sources, major phytochemical classes, key physicochemical properties, anticancer mechanisms, and formulation challenges.

Key Botanical Source	Phytochemical Name	Major Phytochemical Class	Key Physicochemical Properties	Formulation Challenges
*Vitis vinifera*	Resveratrol	Polyphenols	Molecular weight: 228.24 g/mol Log P: 3.1 (moderately lipophilic) Solubility: Less soluble in water, soluble in ethanol and other organic solvents	Poor aqueous solubility, rapid metabolism, sensitive to light, heat, oxygen, and pH
*Curcuma longa*	Curcumin	Polyphenols	Molecular weight: 368.38 g/mol Log P: 3.29 Solubility: Very poor water solubility, soluble in ethanol, DMSO, and acetone	Poor aqueous solubility, unstable under physiological pH, sensitive to light, heat, and oxidative conditions
*Berberis aristata*	Berberine	Alkaloids	Molecular weight: 336.36 g/mol Log P: 1.5 (moderate hydrophilicity) Solubility: Poor water solubility, soluble in acidic aqueous solutions and alcohols	Low water solubility, sensitive to light and heat, rapid metabolism and exhibits poor intestinal absorption
*Catharanthus roseus*	Vincristine/Vinblastine	Alkaloids	Molecular weight: 824.98 g/mol/811.04 g/mol Log P: 1.7/3.3 Solubility: Sparingly soluble in water, more soluble in acidic aqueous and organic solvents	Poor aqueous solubility, sensitive to elevated temperature and light, hydrolyses in alkaline pH
*Taxus brevifolia*	Paclitaxel	Alkaloids	Molecular weight: 853.91 g/mol Log P: 3.96 Solubility-Insoluble in water, soluble in organic solvents like Cremophor EL, ethanol, DMSO	Low water solubility, prone to degradation under extreme pH, light, and heat, high toxicity
*Camellia sinensis*	Epigallocatechin-3-gallate (EGCG)	Polyphenol	Molecular weight: 458.7 g/mol Log P: 0.1 Solubility: Soluble in water, acetone, ethanol, pyridine, tetrahydrofuran, and methanol	Unstable at neutral and alkaline pH, degrades upon exposure to light, heat, and oxygen, poor absorption
*Glycine max*	Genistein	Alkaloids	Molecular weight: 270.24 g/mol Log P: 2.62 (moderately lipophilic) Solubility: Poorly soluble in water, more soluble in organic solvents like ethanol and DMSO	Low aqueous solubility, sensitive to light, heat, oxygen, and pH, unstable under alkaline conditions
*Camptotheca acuminata*	Camptothecin	Alkaloids	Molecular weight: 348.35 g/mol Log P: 1.71 Solubility: Very poor water solubility, soluble in DMSO and ethanol	Low water solubility, unstable at neutral and basic pH
*Quercus robur*	Quercetin	Polyphenols	Molecular weight: 302.24 g/mol Log P: 1.82 Solubility: Poorly soluble in water, soluble in alcohol and alkaline solutions	Poor aqueous solubility, rapid metabolism, unstable in alkaline conditions, sensitive to light and oxidation
*Nigella sativa*	Thymoquinone	Terpenoids	Molecular weight: 164.20 g/mol Log P: ~2.54 Solubility: Insoluble in water, soluble in ethanol, methanol, chloroform, DMSO, and acetone	Poor aqueous solubility, Unstable in aqueous medium, sensitive to light, heat, and oxidation, and metabolic instability
*Beta vulgaris*	Betalains	Betacyanins and Betaxanthins	Molecular weight: 550 g/mol Log P: −3.1 Solubility: Highly soluble in water, insoluble in organic solvents	Poor stability under light, heat, and oxygen exposure, degradation at neutral or alkaline pH, limited lipophilicity, restricting incorporation into lipid-based systems, susceptibility to enzymatic and oxidative degradation during processing and storage
*Rosmarinus officinalis*	Ursolic acid	Triterpenoids	Molecular weight: 456.70 g/mol Log P: 7.09 Solubility: Very poor water solubility, soluble in organic solvents like ethanol, DMSO	Poor water solubility, very high lipophilicity, rapid metabolism, and poor absorption
*Brassica oleracea*	Sulforaphane	Isothiocyanates	Molecular weight: 177.28 g/mol Log P: 0.23 Solubility: Soluble in methanol, ethanol, DMSO, or ethyl acetate, water insoluble	Poor aqueous solubility, stability issues, short half-life, poor lipophilicity, rapid metabolism
*Plumbago zeylanica*	Plumbagin	Naphthoquinones	Molecular weight: 188.18 g/mol Log P: 2.26 Solubility: Low water solubility, soluble in organic solvents like ethanol and DMSO	Low water solubility, prone to oxidation, sensitive to light and air, and potential toxicity
*Solanum lycopersicum*	Lycopene	Carotenoids	Molecular weight: 536.89 g/mol Log P: 17.6 (extremely lipophilic) Solubility: Insoluble in water, soluble in oils, organic solvents such as chloroform, hexane	Low aqueous solubility, poor absorption, highly sensitive to light, heat, and oxygen
*Tabebuia avellanedae*	β-Lapachone	Quinones	Molecular weight: 242.23 g/mol Log P: 2.35 Solubility: Poorly soluble in water, soluble in DMSO, ethanol, and other organic solvents	Poor aqueous solubility, unstable in aqueous environment, sensitive to light and oxygen

**Table 2:** Comparative overview of various nanocarrier systems based on their composition, encapsulation efficiency, stability, biocompatibility, targeting ability, scale-up potential, and biomedical applications.

Nanocarrier System	Composition	Encapsulation Efficiency	Stability	Biocompatibility	Targeting Ability	Scale-Up Potential	Applications
Solid Lipid Nanoparticles (SLNs)	Solid lipids, surfactants, cosurfactants	High for lipophilic drugs	Good	High	Moderate	Good	Drug and phytochemical delivery, cancer therapy
Nanostructured Lipid Carriers (NLCs)	Solid lipids, liquid lipids, surfactants, cosurfactants	High	Better than SLNs	High	High	Good	Drug and phytochemical delivery, chronic diseases
Nanoemulsions	Oil, water, surfactants, cosurfactants	Moderate to high	Low	High	Low	Good	Rapid drug delivery
Liposomes	Phospholipids, cholesterol, charged lipids (optional), hydration medium	Moderate	Moderate	High	Moderate	Challenging	Vaccines, drugs, and gene delivery
Niosomes	Non-ionic surfactants, cholesterol, charged inducers (optional), hydration medium	Moderate	Moderate	High	Moderate	Challenging	Topical and systemic delivery
Cubosomes	Lipids, stabilizers, aqueous phase	High	Good	High	High	Moderate	Dermal delivery
Ivosomes	Phospholipids, ionic liquids, stabilizers (optional), aqueous phase	Moderate	Limited data	Limited data	Moderate	Low	Emerging drug delivery
Ethosomes	Phospholipids, ethanol, water, optional additives (cholesterol, Propylene glycol, or isopropyl alcohol)	High	Low to moderate	High	High	Moderate	Transdermal drug delivery
Transfersomes	Phospholipids, edge activators (surfactants), optional additives (Ethanol or glycerol, Cholesterol), aqueous phase	High	Low	High	High	Moderate	Transdermal and systemic delivery
Transethosomes	Phospholipids, ethanol, edge activators (surfactants), optional additives (Cholesterol or Propylene glycol), aqueous phase	High	Low	High	High	Moderate	Transdermal and systemic delivery
Inorganic Nanoparticles	Metal and Metal oxides, Silica, Carbon nanotubes, Graphene oxide, Fullerenes, Quantum dots, etc.	Variable	High	Variable	High	Good	Bioimaging, biosensing, diagnostics, and cancer therapy
Polymeric Nanoparticles	Natural/synthetic polymers, crosslinking agents	High	Good	High	High	Good	Sustained release
Dendrimers	Branched synthetic polymers, surface modifiers	Very high	Good	Moderate to high	High	Moderate	Gene delivery, diagnostics
Polymeric micelles	Amphiphilic block copolymers (e.g., PEG-PLA, PEG-PCL)	Moderate to High	Good	Good	Good	Moderate	Poorly soluble anticancer drugs and phytochemicals, and diagnostics

**Table 3:** Typical structural components of solid lipid nanoparticles.

Structural Component	Examples
Solid Lipids	Behenic acid, Beeswax, Carnauba wax, Cetyl palmitate, Glyceryl behenate (Compritol 888 ATO), Glyceryl caprate, Glyceryl monooleate, Glyceryl monostearate (Imwitor 900), Glyceryl palmitostearate (Precirol ATO 5), Hard fat, Hydrogenated vegetable oils, Labrafil M1944, Miglyol 812, Monostearin, Oleic acid, Palmitic acid, Paraffin, Polyethylene glycol (PEG) monostearate, Stearic acid, Tricaprin, Trilaurin, Trimyristin (Dynasan 114), Tripalmitin (Dynasan 116), Tri-stearin (Dynasan 118), Tristearin, Witepsol.
Emulsifiers	Butanol, Butyric acid, Cetylpyridinium chloride, Cremophor EL, Eumulgin SML 20, Lecithin, PEG-40 hydrogenated castor oil, Poloxamer 188, Poloxamer 407, Polysorbate 20, Polysorbate 60, Polysorbate 80, Polyvinyl alcohol, Sodium cholate, Sodium deoxycholate, Sodium dodecyl sulfate, Sodium glycocholate, Sodium oleate, Solutol HS 15, Span 20, Span 60, Span 80, Taurodeoxycholic acid sodium, Tyloxapol, Tween 20, Tween 60, Tween 80.
Co-Surfactants	Ethanol, Glycerol, PEG-400, Propylene glycol, Sodium glycocholate, Sodium taurocholate, Transcutol P.
Cryoprotectants	Glucose, Mannitol, Sorbitol, Sucrose, Trehalose.
Charge Modifiers	Dimethyldioctadecyl ammonium bromide, Dodecylamine (cationic), Sodium stearate (anionic), Stearylamine (cationic).
Penetration Enhancers	Ethanol, Linoleic acid, Menthol, Oleic acid.
Targeting Ligands	Aptamers, Folic acid, Monoclonal antibodies, Peptides, Transferrin.
Polymer Coatings	Chitosan, Hyaluronic acid, PEG, Polyvinyl alcohol.

**Table 5:** Typical structural composition of nanostructured lipid carriers.

Solid Lipid	Liquid Lipid	Surfactant
Beeswax	Caprylic/capric triglyceride	Lecithin
Cetyl palmitate	Carvacrol	PEG 400
Glyceryl behenate (Compritol^®^ 888 ATO)	Crodamolt glyceryl tricaprylate/caprate liquid	Pluronic F127
Glyceryl monostearate	Long-chain triglycerides	Poloxamer 188
Myristyl myristate	Medium chain triglyceride	Polysorbate 80
Glyceryl palmitostearate (Precirol^®^ ATO-5)	Miglyol 812N	Sodium lauryl sulfate
Stearic acid	Oleic acid	Span 80
Tripalmitin	Squalene	Tween^®^ 60
Tristearin	Triglyceride esters	Tween^®^ 80

**Table 6:** Summary of anticancer phytochemicals used in NLC delivery, including preparation techniques, formulation composition, and key findings.

Name	Preparation Method	Composition	Therapeutic Application	Highlights	Reference
Cinnamon, Sage, and Thyme essential oils	Emulsification-ultrasonication	Shorea butter, poloxamer 188	Prostate cancer	Three NLC formulations incorporating cinnamon, sage, or thyme essential oils, optimized using a 2^3^ factorial design, demonstrated excellent structural integrity and good stability at 25 °C for over a year. The NLCs were biocompatible in vitro with normal prostate (PNT2) cells and in vivo in chicken embryos. In prostate cancer (PC3) cells, the NLCs inhibited proliferation and migration and altered cell morphology. In a chicken embryo xenograft model, they suppressed tumor growth and angiogenesis.	[228]
Silymarin	Hot melt emulsification	Compritol ATO 888/Miglyol 812 N, Tween 80	Oral cancer	Silymarin, a poorly water-soluble compound, was successfully encapsulated in NLCs with a particle size of ~316 nm, a PDI of 0.341, and 71% encapsulation efficiency. The developed gel demonstrated sustained drug release and enhanced buccal retention. Compared to plain compound and NLCs, the gel showed a lower IC_50_ value against KB oral cancer cells, indicating greater cytotoxicity due to increased reactive oxygen species generation and apoptosis at the Sub-G0 phase.	[229]
Salvianolic acid	Emulsification-solvent evaporation method	Myrj 52, Lecithin, DSPE-PEG2000-E-[c(RGDfK)^2^]), Glycerol behenate, MCT 812, DSPE-PEG2000-Folate	Antitumour	Dual-targeted NLCs co-loaded with doxorubicin and salvianolic acid demonstrated high encapsulation efficiency (>80%) and small particle size (~18 nm). Surface modification with E-[c(RGDfK)_2_] and folic acid enabled effective targeting of various tumor cells. This formulation showed the strongest anti-tumor effects in vitro and in vivo. Polyphenolic acid mitigated doxorubicin-induced nephrotoxicity, reducing creatinine levels by 61.64% (free form) and 42.47% (NLCs). The E-[c(RGDfK)_2_]/FA modification further reduced kidney toxicity by 46.35% compared to the unmodified NLC-salvianolic acid/doxorubicin group.	[230]
Curcumin	High shear hot homogenization	Glyceryl monooleate, Geleol, Olive oil, Tween 80, Lecithin	Breast cancer	Curcumin-loaded NLCs prepared with glyceryl monooleate demonstrated faster drug release and significantly higher anticancer activity compared to NLCs prepared with Geleol™ and free curcumin under both light and dark conditions. The enhanced cellular uptake was attributed to their small particle size, spherical morphology, and negative zeta potential. Furthermore, glyceryl monooleate contributed to the inhibition of P-glycoprotein expression, thereby enhancing the cytotoxic effects of curcumin.	[227]
Calycosin	Nano-template engineering approach	Miglyol, stearic acid, Tween 80, Span 60, PEG 400, Sucrose stearate	Breast cancer	Calycosin-loaded NLCs exhibited nanoparticle size (100 nm), low PDI (0.27), negative zeta potential (−24.5 mV), spherical morphology, high encapsulation efficiency (89%), and sustained drug release. In vitro studies on MDA-MB-231 cells revealed enhanced apoptosis and dose- and time-dependent cytotoxicity, while in vivo evaluations confirmed significant antitumor activity through biochemical and immunohistochemical analyses.	[231]

**Table 7:** Typical preparation methods utilized for solid lipid nanoparticles and nanostructured lipid carriers.

Method	Procedure	Mechanism	Advantages	Limitations	Formulation/Processing Factors	Reference
Double Emulsion Method	A water-in-oil-in-water (w/o/w) multiple emulsion is formed by dispersing a primary water-in-oil (w/o) emulsion stabilized with a hydrophobic emulsifying agents into an external aqueous phase that contains a hydrophilic surfactant. Nanoparticles are then formed through continuous stirring and solvent evaporation.	Solvent evaporation results in emulsion solidification and lipid crystallization.	Best suited for hydrophilic and peptide-based drugs. Surface alteration of nanocarriers is feasible with water soluble polymers	Requirement of multiple steps, prone to instability (particle coalescence), and low encapsulation efficiency.	Type and concentration of surfactants, phase-volume ratio/Stirring rate, duration, and solvent evaporation conditions.	[244]
High-Pressure Homogenization (Hot and Cold Methods)	Hot: Molten lipids are blended with active ingredients and emulsified with a heated aqueous surfactant solution. This mixture is then subjected to high-pressure homogenization (400–800 bar), generating high-velocity streams (>25 m/s) and intense turbulence to form nanoparticles. Cold: Phytochemical is dispersed in molten lipids, rapidly cooled, and ground into microparticles (50–100 µm). These are then mixed with a cold aqueous stabilizer and homogenized at room temperature to maintain drug stability and minimize drug partitioning into the aqueous phase.	Combination of mechanical shear, cavitation, and turbulence disrupts larger particles, leading to the formation of a stable submicron dispersion.	Produces small particles (<500 nm), high stability, no organic solvents, ideal for thermostable phytochemicals, aseptic processing, ease of scale-up, and low risk of product contamination	High energy and temperature input can degrade thermolabile phytochemicals, aggregation risk, generation of supercooled melts, diverse colloidal structures, and phytochemical partitioning into the water phase	Type and concentration of surfactants, lipid, and stabilizers, phytochemical-to-lipid ratio/Homogenization pressure and number of cycles, pre-emulsification conditions, and cooling rate	[123,245]
Membrane Contractor Technique	Melted lipid phase forced through membrane pores maintained above its melting temperature. Formed droplets are then carried into an aqueous surfactant solution flowing tangentially to the membrane, followed by cooling to ambient conditions, results in the formation of SLNs.	Spontaneous emulsification is initiated at the interface of the membrane due to interfacial tension gradients	Continuous, scalable process, particle size can be controlled based on flux through the membrane	Many process parameters and formulation variables, risk of membrane clogging, and high cost	Type and concentration of surfactant, lipid melting point and viscosity/Membrane pore size, operating pressure, tangential flow rate of aqueous phase, and cooling rate	[246]
Microemulsion Method	Melted lipid is mixed with a surfactant and co-surfactant aqueous solution in a specific ratio, forming a microemulsion when dispersed and diluted with cold water (1:25 to 1:100) under stirring.	Negative surface free energy, driven by a significant decrease in interfacial tension and significant entropy gain during mixing, leads to a spontaneous and thermodynamically stable nano-sized dispersion	Thermodynamically stable, high encapsulation efficiency and low energy method.	Requires high surfactant/co-surfactant concentrations and a large water volume, which may require further processing steps to obtain a concentrated product.	Surfactant/Co-surfactant type and ratio, lipid concentration, oil to water ratio, drug-lipid-ratio/Stirring rate and duration	[247,248,249]
Phase Inversion Temperature Method	The emulsion is heated to the phase inversion temperature, where surfactant affinity for oil and water is balanced, then rapidly cooled to form small, stable droplets.	The method relies on temperature-dependent changes in nonionic surfactants, where heating to the critical temperature balances surfactant affinity for oil and water	Low energy process, requires limited amount of surfactant, uniform sized nanodroplets, highly stable SLNs and economical	Several temperature cycles required, stability affected by cooling rate.	Type and concentration of non-ionic surfactant, oil to water ratio, phytochemical to lipid ratio/Heating rate and phase inversion temperature, number of heating and cooling cycles	[250,251]
Solvent Emulsification/Evaporation	Lipids and phytochemicals are dissolved in an organic solvent, emulsified in an aqueous phase, and nanosized through high-speed homogenization. Vacuum evaporation (Rotavapor) removes the solvent, causing nanoparticle precipitation.	Emulsification followed by evaporation of organic solvent leads to precipitation of lipid nanoparticles.	Suitable for hydrophobic and thermolabile drugs, uniform size distribution.	Insolubility of lipids in organic solvents, residual solvent toxicity concerns, environmental issues, and required additional drying or ultrafiltration processing	Surfactant type and concentration, type of organic solvent, solvent to water ratio, type and concentration of lipid, phytochemical to lipid ratio/Emulsification technique and speed	[252,253]
Solvent Injection Method	Lipid and phytochemical are dissolved in a water-miscible organic solvent and injected rapidly into an aqueous surfactant solution under continuous mechanical agitation. Obtained coarse emulsion is nanosized via high-speed homogenization, followed by vacuum evaporation (Rotavapor) to remove the solvent, leading to nanoparticle precipitation.	Solvent diffusion from the lipid to the aqueous phase, combined with interfacial cavitation and vibration, results in the formation of nanosized lipid nanoparticles.	Simple and fast process, simple equipment, and avoids toxic organic solvents	Limited scalability, residual solvent removal needed.	Organic solvent type, surfactant and lipid type and concentration, phytochemical to lipid ratio/Injection rate, stirring rate and speed, aqueous phase temperature, solvent removal conditions	[254]
Sonocrystallization (Ultrasound-Assisted Method)	Lipid and phytochemical mixture is sonicated in an aqueous surfactant solution, causing cavitation, which leads to the formation of nanoparticles.	Ultrasound energy breaks lipid droplets into smaller particles.	Uniform nanoparticles, suitable for heat-sensitive drugs.	Potential probe contamination, not easily scalable.	Surfactant and lipid type and concentration, drug to lipid concentration/Sonication time and intensity, and crystallization conditions	[255]
Supercritical Fluid Technology	Lipid and phytochemical dissolved in supercritical CO_2_ under pressure expanded rapidly by spraying through a nozzle or atomizer to form nanoparticles.	Rapid expansion results in escape of gas, leads to particle precipitation.	Elimination of organic solvents, broad miscibility of lipids with gases, and the ability to produce SLNs in dry powder form.	Expensive equipment, high operational cost.	Type of supercritical fluid, lipid/drug solubility in supercritical fluid/Nozzle design and diameter, pressure and temperature, expansion rate, drying method	[180,256]

**Table 8:** In vitro and preclinical evaluation techniques for anticancer phytopharmaceutical SLNs and NLCs formulations.

Assessment Category	Evaluation Parameters	Primary Tools/Methods	Significance	Reference
Particle size analysis	Size range (nm) and polydispersity index (PDI)	Dynamic light scattering (Zetasizer)	Nanoparticles with 10–200 nm size range are ideal for passive targeting of tumor tissue via the EPR effect. PDI < 0.3 indicates uniform particle size, reducing the risk of aggregation or phase separation during storage	[257]
Surface potential	Surface charge (mV)	Dynamic Light Scattering (Zetasizer)	Values > ±30 mV prevent aggregation and indicate good electrostatic stability	[258]
Surface morphology	Shape, surface texture, aggregation/Clustering Core–shell Structure (TEM) Crystallinity/amorphous nature (TEM) 3D Surface topography (AFM)	Scanning electron microscopy (SEM), Transmission electron microscopy (TEM), or Atomic force microscopy (AFM)	Influence biological performance, release behavior, and stability	[259]
Phytochemical encapsulation and loading efficiency	Encapsulation efficiency (%) = Total phytochemical-Free phytochemical/Total phytochemical) × 100 Loading capacity (%) = (Encapsulated phytochemical/Total weight of nanoparticles) × 100	Ultraviolet spectroscopy (UV) High-performance liquid chromatography (HPLC) Centrifugation or ultrafiltration	High encapsulation efficiency and loading capacity ensure minimal phytochemical loss, maximized therapeutic output, and reduced toxicity	[111]
In vitro release studies	Cumulative phytochemical release, release kinetics	Franz diffusion system, dialysis setup with artificial membrane (cellulose or dialysis membrane)	Predicts in vivo behavior in physiological and tumor-specific environments.	[260]
Passive permeation	Flux, permeability coefficient, lag time	Franz diffusion cell, permeability chambers, side-by-side diffusion chambers with artificial (PAMPA, Caco-2) or biological membrane (excised tissues)	Assess the performance of phytochemical transport across biological membranes to help predict in vivo therapeutic outcomes	[261]
Long-Term Stability	Monitor formulation stability over time by assessing various in vitro characterization parameters	Stability chambers (temperature and humidity, oxidative stress chambers, photostability chambers	Retain their therapeutic potency, physicochemical integrity, and safety over time	[262,263,264]
Reactive Oxygen Species (ROS) generation	Fluorescence Intensity, % ROS-positive cells, mean fluorescence intensity, time and dose dependent ROS production, ROS source specificity	Microplate reader, flow cytometer, or fluorescence microscope after staining with ROS-sensitive dyes., 2′,7′-Dichlorodihydrofluorescein diacetate (DCFH-DA), use of N-Acetylcysteine (NAC) or Mitochondria-targeted hydroethidine (MitoSOX)	Provide mechanistic insight into ROS-mediated cytotoxicity, validate enhanced intracellular delivery, trigger apoptosis or autophagy, support combination therapy design, and early biobarker for therapeutic response	[265]
Protein/Gene expression analysis	Evaluation parameters include the ratio of gene/protein expression in treated cells versus control (fold change), relative quantification, band intensity, protein concentration, expression of key pathway markers including apoptosis (Bax, Bcl-2, caspase-3, PARP), autophagy (LC3-II, Beclin-1, p62/SQSTM1), cell cycle regulation (Cyclins, CDKs, p21, p53), oxidative stress (Nrf2, HO-1, SOD), drug resistance (P-gp, MRP1, ABC transporters)	qRT-PCR or RT-PCR, microarray analysis, RNA-Seq, Western blotting, ELISA, Immunofluorescence/Immunocytochemistry (IF/ICC), Flow cytometry, Mass spectrometry-based proteomics	Reveals molecular mechanism of action, confirms pathway-specific targeting, supports selectivity and safety, guides in vivo translation and biomarker identification	[266]
Cytotoxicity assay	IC50 value, percentage cell viability, time and dose-dependent cytotoxicity	MTT/XTT/MTS, Resazurin (Alamar Blue), Tryptan blue Exclusion, LDH release	Evaluate the anticancer potential by measuring cell viability and potency (e.g., IC_50_), and provide initial insights into cytotoxic effects	[267]
Pharmacokinetics and Biodistribution mapping	Drug concentration in tissues, tumor-to-organ ratio, plasma drug concentration (Cmax, Tmax, t½), biodistribution profile (graphical/heatmap), fluorescence or radioactivity intensity, organ accumulation index	Fluorescence imaging (DiR, FITC-labeled), radiolabel tracking, quantification (HPLC, LC-MS/MS), imaging (MRI, PET, SPECT)	Evaluates site-specific delivery, validates nanoformulation efficacy and safety, correlates therapeutic outcomes, and guides route and dosage optimization	[268]
Animal models (xenograft and synergistic) of cancer	Tumor volume and weight, tumor growth inhibition, survival rate/median survival time, tumor doubling time, histopathological and hematological examination	Digital vernier calipers, bioluminescence imaging, ultrasound/MRI/PET-CT, hematoxylin and eosin staining, immunohistochemistry, TUNEL assay, fluorescence/confocal microscopy, markers of organs such as liver (ALT, AST), and kidney (creatinine, BUN)	Validating therapeutic efficacy and safety, revealing molecular mechanisms, mapping biodistribution, preclinical validation before human clinical trials.	[269]
Cellular Uptake	Uptake efficiency, intracellular localization, mean fluorescence intensity, mechanisms of uptake, concentration and time dependent uptake	Fluorescent microscopy, flow cytometry, confocal imaging, clathrin-mediated, caveolae-mediated, or micropinocytosis inhibitors	Confirm effective phytochemical delivery, predicts therapeutic efficacy, guides formulation optimization and mechanistic understanding	[270,271,272]
Mitochondrial Membrane Potential (ΔΨm) Assay	Fluorescence intensity, red/green fluorescence ratio, percentage of cells with depolarized mitochondria, time and dose dependent response	JC-1, Rhodamine 123 dyes, flow cytometry	Early detection of apoptosis, assessment of mitochondrial health, mechanistic insights into cytotoxicity and screening of mitochondrial targeting compounds	[273]
Apoptosis/Necrosis Assay	Differentiate between apoptosis and necrosis via Annexin V-FITC/Propidium Iodide (PI) staining, Percentage of cells in each quadrant, and mean fluorescent intensity	Annexin V-FITC/PI staining, flow cytometry Caspase-3/7 activity assay, TUNEL assay, DAPI or Hoechst staining, Western blot for Bax, Bcl-2, cleaved PARP	Confirms efficacy of nanoformulations, mechanism of cell death, validates target action on cancer cells, supports dose optimization and safety	[274,275]
Autophagy Assay	LC3-II expression levels, LC3 puncta formation, Autophagic flux, p62/SQSTM1 levels, Acridine orange (AO) or monodansylcadaverine (MDC) staining, Beclin-1 expression	Immunofluorescence microscopy (LC3-GFP or LC3-mCherry), lysosomal inhibitors (e.g., bafilomycin A1, chloroquine), fluorescence microscopy, flow cytometry, RT-qPCR	Reveals autophagy’s role in cell survival or cell death, elucidates mechanism of action, predicts drug resistance or sensitization, guides combination strategies with autophagy enhancers or inhibitors	[276]

**Table 9:** Patent applications related to anticancer phytochemicals embedded in solid lipid nanoparticles.

Application ID	Publication Date	Title	Summary of Invention
201811376407.9	26 May 2020	Preparation method of folic acid-targeted silymarin solid lipid nanoparticles	A folic acid-modified silymarin solid lipid nanoparticle system was developed to enhance lung tumor targeting. The formulation involves conjugating silymarin-loaded SLNs with folic acid–PEG3350–cephalin, enabling selective delivery to tumor cells. This targeted approach improves silymarin’s bioavailability, reduces toxicity, enhances therapeutic efficacy, and supports early patient recovery. The method is simple, economical, and environmentally friendly, making it suitable for clinical application.
202010890499.3	18 December 2020	Preparation method and application of quercetin (QT) and MicroRNA-150 co-loaded cationic solid lipid nanoparticles (SLNs)	Describes a method for preparing cationic SLNs co-loaded with quercetin and microRNA-150. The resulting nanoparticles exhibit good stability, biocompatibility, and effectively deliver both into HUVEC cells.
202111029614	1 July 2021	A formulation of hesperidin containing solid lipid nanoparticles through oral route and methods thereof	Hesperidin-loaded SLNs were developed using cold homogenization and ultrasonication to improve hesperidin’s poor solubility and low bioavailability. The formulation was evaluated for particle size, entrapment efficiency, drug content, diffusion, and morphology, aiming to enhance its effectiveness for oral delivery.
17439617	19 May 2022	Solid lipid nanoparticles of curcumin	Discloses a method for preparing curcumin-loaded SLNs with particle sizes ranging from 20 to 800 nm and exhibits high entrapment efficiency (50–100%).
202210290513.5	28 June 2022	Indirubin solid lipid nanoparticles and preparation method thereof	Indirubin-loaded solid lipid nanoparticles were developed using biocompatible materials to enhance the drug’s solubility, membrane permeability, and oral bioavailability. The formulation consists of indirubin (1–5%), lipid material (85–95%), and an emulsifier (3–10%), offering an improved delivery system for this traditional Chinese medicine compound.
3207752	11 August 2022	Process for preparing nanoformulation for delivery of berbamine	This invention describes a simple and efficient method for preparing berbamine-loaded solid lipid sustained-release nanoparticles. By adjusting the pH of the aqueous or lipid phase during formulation, the process achieves a high drug loading (12–50% *w*/*w*) and entrapment efficiency above 90%.
202211064052	18 November 2022	Resveratrol loaded nanoparticles and preparation method thereof	The formulation is prepared by dissolving resveratrol, soya phosphatidylcholine S-100, and tristearin in a chloroform-methanol mix, followed by emulsification, sonication, solvent evaporation, and purification through centrifugation.
202221069827	30 December 2022	Naringenin loaded solid lipid nanoparticles for oral delivery	Naringenin-loaded SLNs were developed using compritol to enhance its low oral bioavailability. Optimized using response surface methodology, the SLNs showed a particle size of 66.56 nm, good stability, and a sustained drug release of 94.97% over 24 h. Pharmacokinetic evaluation in rats demonstrated a 3.1-fold enhancement in bioavailability compared to naringenin suspension, indicating the formulation’s potential for improved oral delivery via intestinal lymphatic transport.

**Table 10:** Overview of updated patent filings on nanostructured lipid carriers loaded with anticancer phytochemicals.

Application ID	Publication Date	Title	Summary of Invention
201110029485.3	27 January 2011	Resveratrol nanostructured lipid carrier and preparation method thereof	This invention presents an NLC formulation containing 0.1–1 wt% resveratrol, 2–20 wt% emulsifier, 2–30 wt% composite lipids (glyceryl triacetate, acetylated monoglyceride, and diisopropyl adipate), and water as the balance. The formulation offers enhanced water solubility, good stability, and is well-suited for use in cosmetic products.
102016000602686	7 December 2016	N-acetyl-L-cysteine modified curcumin nanostructured lipid carrier used for oral administration	This invention relates to an orally administered NLC modified with N-acetyl-L-cysteine for enhanced delivery of curcumin. The formulation includes curcumin, surfactants, lipid components, and N-acetyl-L-cysteine or its derivative. This NLC significantly improves curcumin’s water solubility, promotes its absorption, and enhances its oral bioavailability.
201910145633.4	16 July 2019	Nanostructured lipid carrier (NLC) for collaborative treatment of glioma as well as preparation method and application of NLC	This invention describes NLC formulation containing glyceryl monostearate, triglyceride, temozolomide, curcumin, poloxamer 188, and ethanol, developed via a microemulsion method. The NLC exhibits a uniform particle size (<100 nm), zeta potential of −8.54 ± 0.51 mV, and high entrapment efficiencies for temozolomide (91.53 ± 0.07%) and curcumin (88.64 ± 0.99%).
20828766	12 October 2022	Nanostructured drug delivery system as a multifunctional platform for therapy	This invention describes a functionalized lipid-based nanoplatform for targeted drug delivery, where one or more ligands are attached to the nanoparticle surface to enable specific targeting. The system encapsulates at least one active pharmaceutical ingredient and is designed to enhance the treatment of various diseases, particularly different types of cancer, including glioblastoma.
202311053306	15 September 2023	loaded nanostructured lipid carrier for breast cancer	This invention focuses on the formulation, optimization, and evaluation of quercetin-loaded NLCs. The quercetin-NLCs were successfully fabricated employing hot high-pressure homogenization and demonstrated enhanced drug absorption, protection of quercetin from degradation, extended circulation time, targeted uptake by cancer cells, and reduced systemic toxicity.
202511011758	28 February 2025	A transferrin-conjugated dual drug loaded nanostructured lipid carrier for glioblastoma and a method thereof	This invention describes transferrin-conjugated NLCs co-loaded with docetaxel and quercetin. The formulation is designed for intranasal delivery, enhancing bioavailability, cellular uptake, and therapeutic efficacy. It shows selective uptake by U87-MG glioblastoma cells and synergistic cytotoxicity.

## Data Availability

The data presented in this study is contained within this article.
